# Synthesis of Controllable Cu Shells on Au Nanoparticles with Electrodeposition: A Systematic *in Situ* Single Particle Study

**DOI:** 10.1021/acs.jpcc.2c08910

**Published:** 2023-03-01

**Authors:** Mohsen Elabbadi, Christina Boukouvala, Elizabeth R. Hopper, Jérémie Asselin, Emilie Ringe

**Affiliations:** † Department of Materials Science and Metallurgy, University of Cambridge, 27 Charles Babbage Road, Cambridge, United Kingdom CB3 0FS; ‡ Department of Earth Sciences, University of Cambridge, Downing Street, Cambridge, United Kingdom CB2 3EQ; § Department of Chemical Engineering and Biotechnology, University of Cambridge, West Cambridge Site, Philippa Fawcett Drive, Cambridge, United Kingdom CB3 0AS

## Abstract

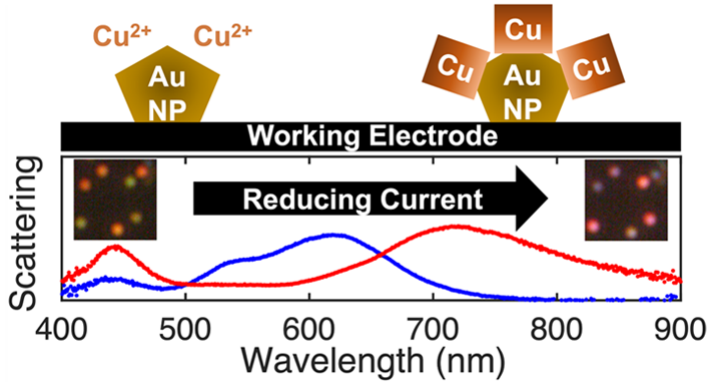

Bimetallic Cu on Au nanoparticles with controllable morphology and optical properties were obtained via electrochemical synthesis. In particular, multilobed structures with good homogeneity were achieved through the optimization of experimental parameters such as deposition current, charge transfer, and metal ion concentration. A hyperspectral dark field scattering setup was used to characterize the electrodeposition on a single particle level, with changes in localized surface plasmon resonance frequency correlated with deposition charge transfer and amount of Cu deposited as determined by electron microscopy. This demonstrated the ability to tune morphology and spectra through electrochemical parameters alone. Time-resolved *in situ* measurements of single particle spectra were obtained, giving an insight into the kinetics of the deposition process. Nucleation of multiple cubes of Cu initially occurs preferentially on the tips of Au nanoparticles, before growing and coalescing to form a multilobed, lumpy shell. Modifying the surface of Au nanoparticles by plasma treatment resulted in thicker and more uniform Cu shells.

## Introduction

Bimetallic, decorated, and core–shell nanoparticle (NP) architectures are central to the creation of multifunctional systems. These systems can combine a variety of attributes, including optical, magnetic, electronic, catalytic, and so on. Combining plasmonic NPs with catalytic surfaces has been of particular interest in the past decade owing to the development and understanding of plasmon-enhanced catalysis.^1−3^ In these systems, the core can sustain light-driven coherent oscillations of its conduction electrons, a size-, shape-, and composition-dependent phenomenon named a localized surface plasmon resonance (LSPR). These oscillations act as antennae for light, whose energy can then be passed on via decay products, such as hot electrons or heat, to a surface catalytic metal where reactions can occur efficiently. Many such architectures have been demonstrated so far, either as core–shells^4,5^ or randomly decorated NPs.^4−6^

A common approach for the synthesis of core–shell or decorated NPs is colloidal reduction, where one reduces one metal to form the core and then in a subsequent step another metal to form a shell or small attached NPs. Alternatively, coreduction techniques, where both metals are in solution at the same time, often result in surface enrichment due to differences in reduction potential.^7^ These differences in reduction potential also offer synthetic opportunities via galvanic replacement,^8^ where a core metal, including plasmonic elements such as Ag,^9^ Cu,^10^ Al,^11^ or Mg,^12−14^ transfers its electrons to a more noble metal such as Au, Pt, or Pd.

In contrast to the fixed potential difference leading to an electron transfer in galvanic replacement, electrodeposition offers a fully controllable potential, as well as the ability to tune the electron transfer rate and the total number of electrons transferred, hence the number of metal ions reduced. Electrodeposition is an important industrial process, although it has received little attention for nanosystems. Interestingly, electrodeposition on nonspherical NPs is expected to produce nonuniform coatings. Indeed, electrochemical etching, essentially the opposite process to electrodeposition, was experimentally shown to preferentially affect the tip of sharp triangles,^15^ consistent with the predicted cathodic shift of the redox potential with decreasing particle radius (small NPs and corners are more oxidation-prone).^16^ This cathodic shift implies that tips are a favorable location for electrodeposition, as also suggested by the preferential submonolayer metal deposition occurring on high order (high Miller index), non-nanoscale surfaces and high curvature regions.^17^ In addition, different crystallographic orientations are known to have different electrochemical potentials.^18−21^ Together, these variations in potential predict a rather complex shape-dependent deposition that opens many opportunities for new architectures.

Few studies of electrochemical control of NPs related to plasmonic applications have been published to date. Early on, the change in electron density upon the application of a potential in the absence of electrodeposition has been shown to change the LSP frequency,^22^ a report later expanded on by Byers et al., who showed the significant heterogeneity of such changes.^23^ Since then, several electrochemical syntheses and nanoscale modification approaches have emerged and have been tracked optically owing to the LSPR’s sensitivity to NP size, shape, and composition. These reports include the electrochemical formation and oxidation of single Ag NPs,^24−26^ oxidation of Ag NPs in the presence of Cl^–^,^27^ photooxidation of Ag,^28^ and electrochemical Hg amalgamation on Au NPs.^29,30^

While these electrochemical approaches allowed for significant progress to be made in the understanding of reduction and oxidation of metallic NPs, only a small number of works have interrogated the electrochemical formation of bimetallic NPs. Notably, Chirea et al. reported the deposition of Ag onto Au nanostars and correlated *in situ* dark field optical spectroscopy with scanning electron microscopy (SEM).^31^ The authors reported a notable blue shift due to Ag deposition on a few nanostars, and presented images confirming the addition of Ag, paving the way for more systematic studies. Later, Oh et al. tracked optical changes due to the deposition of Cu on Ag across a range of deposition conditions and then observed representative NPs by scanning electron microscopy.^32^ They reported the systematic control of the morphology of Cu on Ag by varying the potential sweep rate, which they hypothesized was due to underpotential deposition, although its direct observation was not possible. Later, Hu et al. used an objective and condenser of the immersion type to enhance the sensitivity of their dark field experiment, leading to the observation of both underpotential and bulk electrodeposition of Ag on Au octahedra and cubes.^33^ Taken together, these previous studies demonstrate the possibility of tracking electrochemical changes at the single particle level; here we aim to use such spectroelectrochemical tools to monitor the electrochemical synthesis of bimetallic NPs with controlled shell thickness and morphology. We focus on the deposition of Cu on Au, both as an easy to handle/analyze model and a realistic catalytic system.^34^

Specifically, we show control of the extent and morphology of Cu electrodeposition on faceted Au NPs, leading to tunable bimetallic nanostructures. Striking changes in plasmonic behavior observed after deposition, correlated with electron micrographs, reveal a homogeneous and reproducible extent of deposition and morphology dependent on, mainly, the deposition current and total charge transfer. Such deposition occurs rapidly at the single NP level, as supported by single particle optical tracking during deposition. Taken together, this approach and understanding of nanoscale electrodeposition provides a well-controlled pathway to produce arbitrary bimetallic NPs and an alternative to colloidal synthesis.

## Methods

### Au NP Synthesis

**Figure 1:**
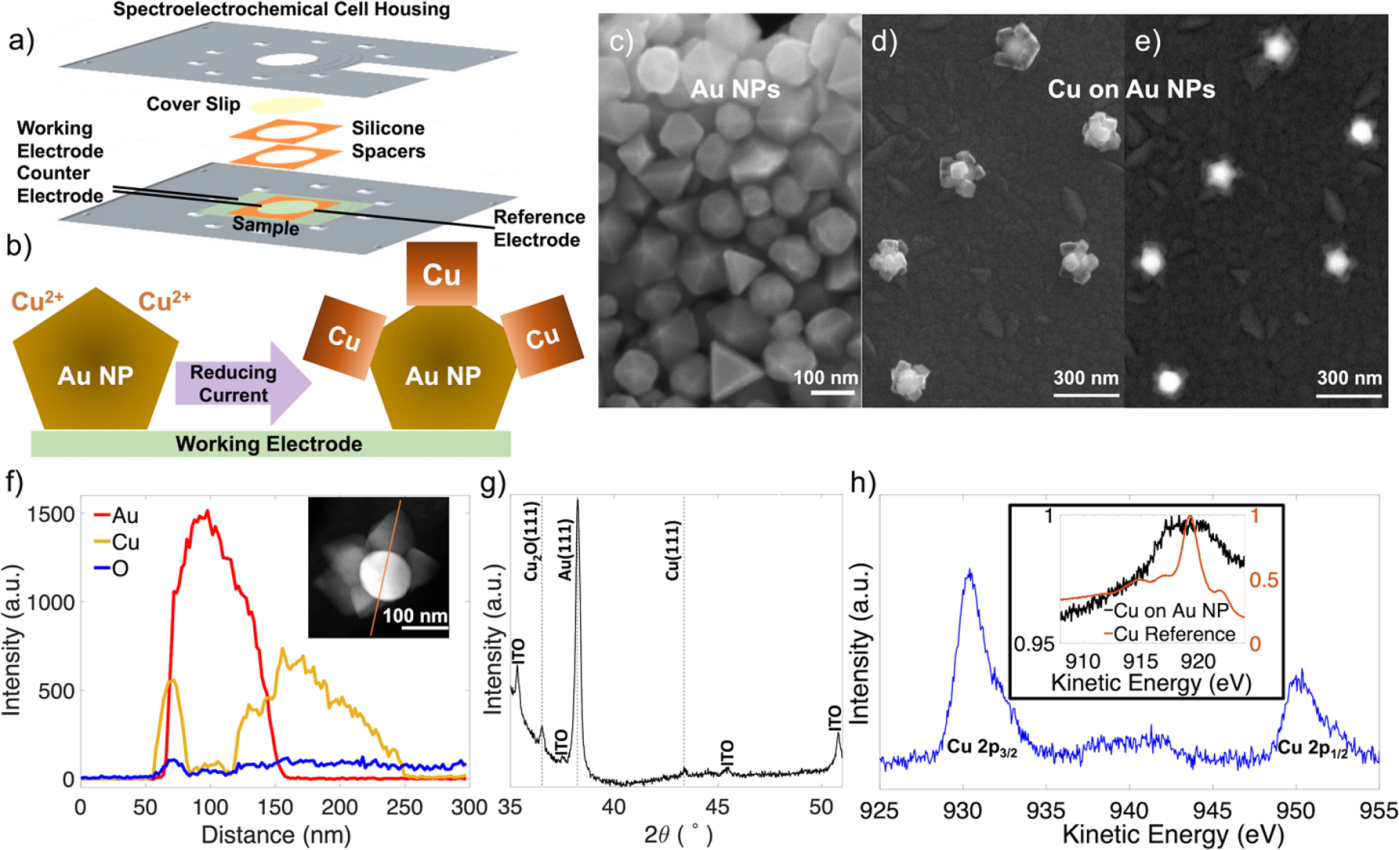
Overview of Cu electrodeposition on Au NPs. (a) Schematic diagram of the spectroelectrochemical cell, (b) schematic of Cu electrodeposition on a single Au decahedral NP, (c) SEM image of the Au NP sample deposited at high density to show its mixture of decahedra, isocahedra, and truncated bitetrahedra, (d) SE and (e) BSE images of Au NPs after Cu electrodeposition at a density appropriate for deposition, (f) STEM-EDS line scan of a Cu on Au NP, where the scan starts below and ends above the NP, (g) XRD pattern of NPs with Cu, Cu_2_O, and substrate (ITO) peaks labeled, and (h) XPS 2p and normalized LMM Auger (inset) spectra of sample and Cu foil for reference.

Tetrachloroaurate trihydrate (HAuCl_4_·3H_2_O, 99.9+%), poly(vinylpyrrolidone) (PVP, M_W_ 55,000), diethylene glycol (DEG, 99%), and tetraethylene glycol (TEG, 99%) were purchased from Sigma-Aldrich and used without further purification. Au NPs were synthesized following a previously published synthesis by Seo et al.,^35^ yielding a mixture of predominantly decahedra, icosahedra, and truncated bitetrahedra (Figure 1). Briefly, 3.5 g of PVP was dissolved in 12.5 mL of DEG and refluxed for 5 min. A solution of 10 mg of HAuCl_4_·3H_2_O in 1 mL of DEG was added to the reaction mixture which was refluxed for a further 10 min. The mixture was cooled to room temperature and diluted with 12.5 mL of ethanol, followed by centrifugation at 6000 rpm for 30 min. Ethanol addition and centrifugation was repeated 4 times.

### Spectroelectrochemistry

A three electrode spectroelectrochemical setup was used to perform and track electrodeposition. Indium tin oxide (ITO) coated slides (SPI supplies, 8–12 Ω resistance) were cleaned by immersion into a 1:1:5 mixture of NH_4_OH (35 wt % solution in water):H_2_O_2_ (30 wt % solution in water):H_2_O at 70 °C for 1 min, followed by rinsing with deionized water. The slides were then decorated by Au NPs by drop casting to form the working electrode. The area of the working electrode was constant at 5.3 cm^2^. A Pt mesh (Alfa Aesar, 100 mesh woven, 0.0762 mm diameter wire, 99.9% purity) was used as the counter electrode. A custom built, 1 mm barrel diameter Ag/AgCl electrode (Alvatek) was used as the reference electrode. All three electrodes were held in solution by a custom cell suitable for an optical microscope stage (Figure 1a). Silicone isolators (Grace Bio-Laboratories, 13 mm diameter opening) acted as spacers in the cell to separate the electrodes and ensure a leak-proof seal. The aqueous electrolyte solution (approximately 270 μL) contained 5 mM CuSO_4_·5H_2_O (Sigma-Aldrich, 98% purity) for galvanostatic measurements and 0.2 mM CuSO_4_·5H_2_O for potential sweep experiments, except for concentration studies where it was varied from 0.04 mM to 25 mM. A supporting electrolyte, Na_2_SO_4_ (Sigma-Aldrich, 99.9%), was present in 40 mM concentration. All electrochemical experiments were conducted with a Gamry Reference 600 potentiostat.

### Dark Field Optical Microscopy

Single particle scattering data was obtained with dark field optical microscopy similarly to what was described in ref (36). The sample was illuminated by a 12 V 100 W halogen lamp (Nikon D-LH/LC) through a 0.95–0.80 numerical aperture (NA) dark field condenser (Nikon), and scattering was collected by a 0.5–1.3 NA objective (Nikon CFI Plan Fluor 100XS oil set at 0.5 NA) in an inverted optical microscope (Nikon Eclipse Ti-2) equipped with a 80/20 beam splitter between the spectrometer and color camera ports. For spectral measurements, light was dispersed by a Princeton Instruments IsoPlane SCT320 spectrometer equipped with a 50 g/mm grating and recorded on a Princeton Instruments ProEM HS 1024 × 1024 EMCCD. A piezoelectric stage (Physik Instrumente P-545.3C7) was used for sample positioning and stage scanning. Time series data were acquired at 0.25 s intervals with the NPs in solution. Hyperspectral measurements were acquired *ex situ* before and after deposition (following rinsing with deionized water and drying) with a step size of 0.3 μm and an acquisition time of 2 s per step. Dark field optical scattering images were obtained with a color camera (Thorlabs CS505CU - Kiralux 5.0 MP Color CMOS Camera).

### Electron Microscopy, X-ray Diffraction and Photoelectron Spectroscopy

SEM was performed with a Quanta-650F FEG-SEM. Acquisition was performed at 15 kV using Everhart–Thornley (ET) and concentric backscattered (CBS) electron detectors. Characteristic X-rays were detected by a Bruker XFlash 6-30 energy dispersive X-ray spectroscopy (EDS) detector. Samples for high-angle annular dark field scanning transmission electron microscopy (HAADF-STEM) were prepared by sonication of the ITO-coated slide in IPA to obtain dispersed NPs, which were subsequently drop cast on Si_*x*_N_*y*_ grids. HAADF-STEM was carried out on a FEI Tecnai Osiris operated at 200 kV; STEM-EDS was performed using a Bruker Super-X quad detector system. X-ray diffraction (XRD) was performed on a Bruker D8 DAVINCI with position sensitive detector and Cu Kα source. X-ray photoelectron spectroscopy (XPS) was performed using a Escalab 250XI spectrometer from Thermo Fisher Scientific with an Al Kα source.

### Data Processing

The optical data acquired from hyperspectral microscopy was analyzed with a home-built MATLAB graphical user interface. Briefly, the *x*–λ data files were stacked to obtain the 3D *x*–*y*–λ datacube. The data were corrected for background, lamp profile, and dark counts as described in ref (36). NPs were identified and localized by setting a threshold value determining an intensity isoline that defines the NP scattering border and its center. Then, intensities were integrated over a 3 × 4 pixel region corresponding to a 0.9 × 0.56 μm area. 80–160 NPs were selected per sample, giving labeled particle spectra. A 2D image suitable for SEM navigation was created by taking a sum along the spectral dimension.

### Numerical Simulations

Scattering cross sections and field distributions were obtained numerically by solving Maxwell’s equations in the discrete dipole approximation using DDSCAT^37^ for nonspherical shapes and via a transfer-matrix method using STRATIFY^38^ for spheres. The frequency dependent refractive indexes of Au and Cu were taken from Johnson and Christy,^39^ while that of Cu_2_O was from Palik.^40^ The ambient refractive index was set to 1. Unless stated otherwise, an orthogonally polarized field direction, forming an angle of 31° to the substrate (substrate not included in the simulations), was used to approximate the unpolarized light and the light cone generated by the dark field condenser of the experimental setup. All calculations were carried out with an interdipole distance of 2 nm, and ParaView was used to visualize the electric fields.

## Results and Discussion

### Morphology of Electrodeposited Cu on Au

Electrodeposition of Cu on Au NPs was performed in a transparent liquid cell and tracked optically. The Au NPs were obtained using a published synthesis,^35^ yielding a mixture of PVP-capped decahedra, isocahedra, and truncated bitetrahedra with an average size of 126 ± 9 nm (tip to edge length, i.e., height), 119 ± 6 nm (tip to tip), and 133 ± 7 nm (tip to edge), respectively (Figure 1c). The NPs were drop cast on indium tin oxide (ITO)-coated glass coverslips with a density of 680 ± 30 diffraction limited spots per mm^2^, and the coverslips were positioned as the working electrode of the spectroelectrochemical cell (Figure 1a). Cu was then electrodeposited from an aqueous solution of CuSO_4_ in the presence of Na_2_SO_4_, a common electrolyte for Cu deposition^41−43^ which provides conditions in which direct reduction of Cu^2+^ to Cu is dominant.^41^ A detailed discussion of the electrodeposition of Cu in Na_2_SO_4_ can be found in ref (44). Electrodeposition in the presence of Cl^–^ was also attempted but yielded white precipitates, making optical tracking difficult.

A typical deposition, with a current of 150 μA and a deposition time of 3.2 s (0.48 mC total charge transfer), yielded bimetallic structures in which Cu nucleated on the corners and edges of Au NPs and formed a multilobed, lumpy shell of similar appearance on all NPs (secondary electron (SE) and backscattered electron (BSE) images, Figure 1d,e). This morphology results from Cu nucleation at multiple sites, with preference for high curvature areas owing to their potential-induced electric field concentration, as observed for Ag on Au.^31^ While quantifying the thickness of such shells is challenging, we estimate, from measuring 30 NPs, a value of 45 nm. In the absence of Cu^2+^ ions, no Cu deposition or optical changes were seen (Figure S1). Further, illumination during deposition did not influence the optical or structural observations (Figure S2), nor did the removal of the PVP coating with NaBH_4_ (Figure S2). This latter lack of surfactant effect is unlike what was observed for Cu on Ag,^32^ possibly due to the different strengths of interactions between PVP and Au/Ag.

The shells appearing during deposition were predominantly Cu. In BSE-SEM as well high angle annular dark field scanning transmission electron microscopy (HAADF-STEM) images, Cu can be readily identified by its lower brightness compared to the higher Z Au (*e.g.*, Figures 1 and S3). Spatially resolved elemental information from STEM energy dispersive X-ray spectroscopy (STEM-EDS) confirmed the lobes are mainly Cu with, in some cases, an oxygen signal tracking that of Cu (Figures 1f and S3). At the bulk level, powder X-ray diffraction (XRD) revealed the presence of both Cu and Cu_2_O; the peaks were broad and weak due to the nanocrystalline nature of the sample (Figure 1g). Further confirmation of the presence of Cu comes from X-ray photoelectron spectroscopy (XPS, Figure 1h), which showed the Cu 2p peak. The lack of a strong satellite near the 2p peak in XPS indicates the absence of CuO and Cu hydroxides, consistent with their absence in XRD. A broad peak in the Auger LMM spectrum (Figure 1h, inset) appeared comprised of peaks from both Cu and Cu_2_O;^45^ while this spectrum cannot be reliably quantified, it is consistent with previous observations. Cu_2_O is likely formed from the oxidation of deposited Cu, as in refs (46 and 47), and deposition of Cu_2_O is unlikely considering the equilibrium diagram of CuSO_4_^48^ and the potential applied; however, we cannot rule out a codeposition of Cu and Cu_2_O based on our data and previously reported results,^49^*vide infra* for further discussion of oxidation processes and time scales.

### Effect of Deposition on the Plasmonic Response

**Figure 2:**
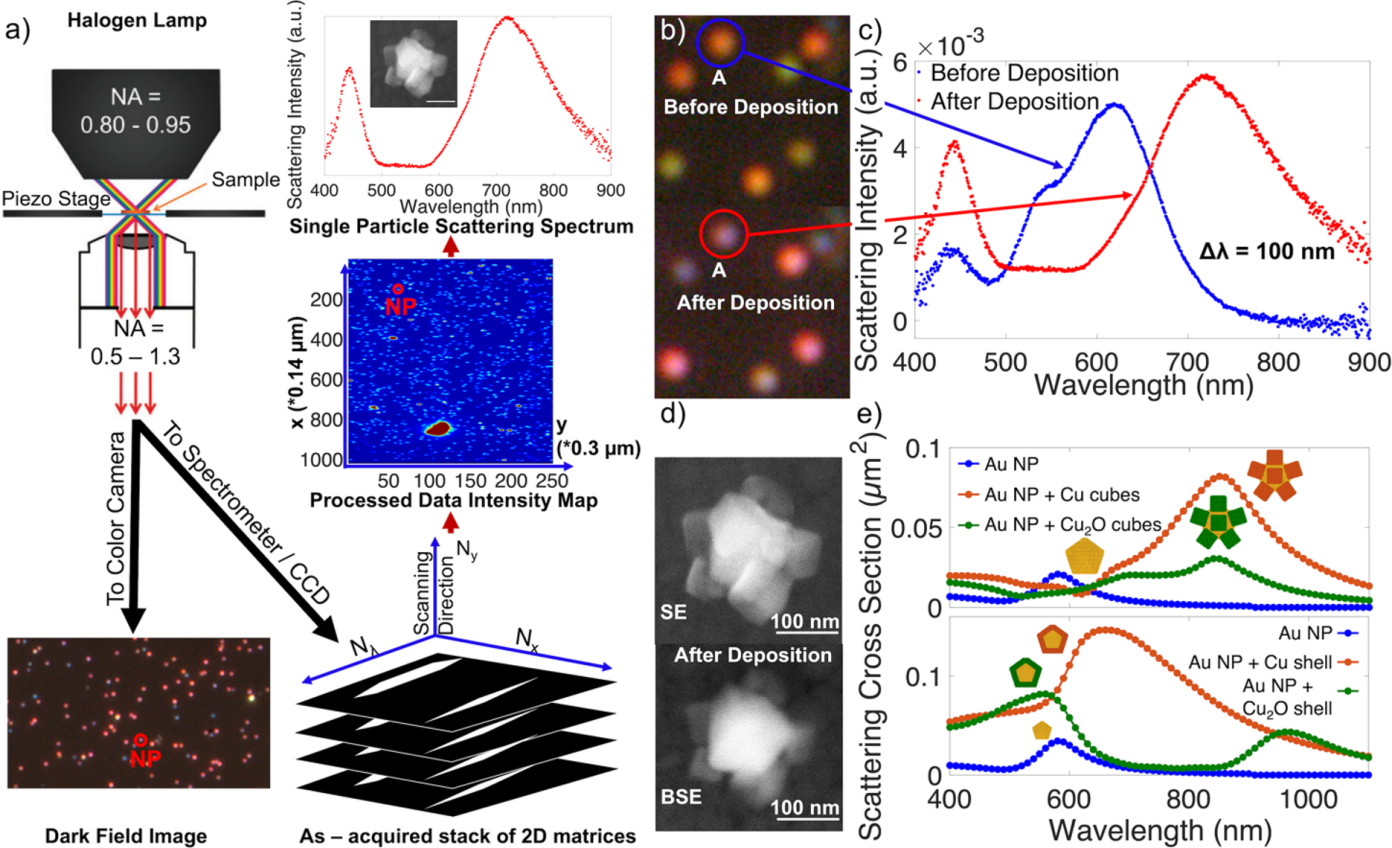
Optical characterization of the electrodeposition of Cu on Au NPs. (a) Schematic of the dark field microscopy setup, showing color camera imaging and hyperspectral data acquisition and processing, as well as correlation between single particle spectra and SEM images, (b) dark field color images before/after deposition, with NP **A** circled, (c) scattering spectrum of NP **A** before/after deposition, and (d) SE and BSE images of NP **A** after Cu deposition. (e) Calculated scattering cross sections for the decahedral Au NP core (edge length 90 nm) decorated with (top) Cu or Cu_2_O cubes of side length 60 nm and (bottom) a conformal Cu or Cu_2_O shell of thickness 40 nm. Dark field microscopy schematic in (a) reproduced from ref (50) with permission. Copyright 2013 Royal Society of Chemistry.

Using a transparent conductive oxide (ITO) to support the Au NPs allowed for the tracking of the LSPR before, during, and after deposition using dark field optical scattering spectroscopy (Figure 2). Here, the scattered light was split 20/80 between a color camera and spectrometer, the former allowing a rapid overview and the latter enabling the acquisition of a hyperspectral data cube via a spatial-scanning (“push-broom”) approach.^36^ From this data cube, we extracted single NP scattering spectra, which were correlated with the NPs’ final morphology obtained with SEM as depicted in Figure 2a.

Optical scattering signatures for a typical deposition of Cu on Au are shown in Figure 2b. To minimize oxidation, hyperspectral measurements before and after deposition were performed in air, with a rinsing and drying step immediately after deposition. The color camera images, showing diffraction limited spots where one or more particles are present, reveal a noticeable change in color after deposition, from green to pink or orange to purple. Accordingly, the optical scattering spectra, obtained at the single particle level, red-shifted significantly after Cu deposition: in Figure 2b, the lowest energy peak, the azimuthal dipole (as assigned in refs (51−53) and shown in Figure S4), for the particle circled in the color image shifts by 100 nm from 618 to 718 nm. Further, we observe, as did others,^51,52^ a shoulder on the high energy side of the azimuthal dipole; this is attributed to the azimuthal quadrupolar LSPR. This quadrupole also shifts; however, quantifying its magnitude is difficult due the low intensity. These shifts are due to the deposition of a shell of Cu-containing crystallites, as observed in the SEM of the same NP (Figure 2d), which redshifts the LSPR by both increasing the plasmonic NP size^54^ and changing its effective dielectric function.^55,56^

**Figure 3:**
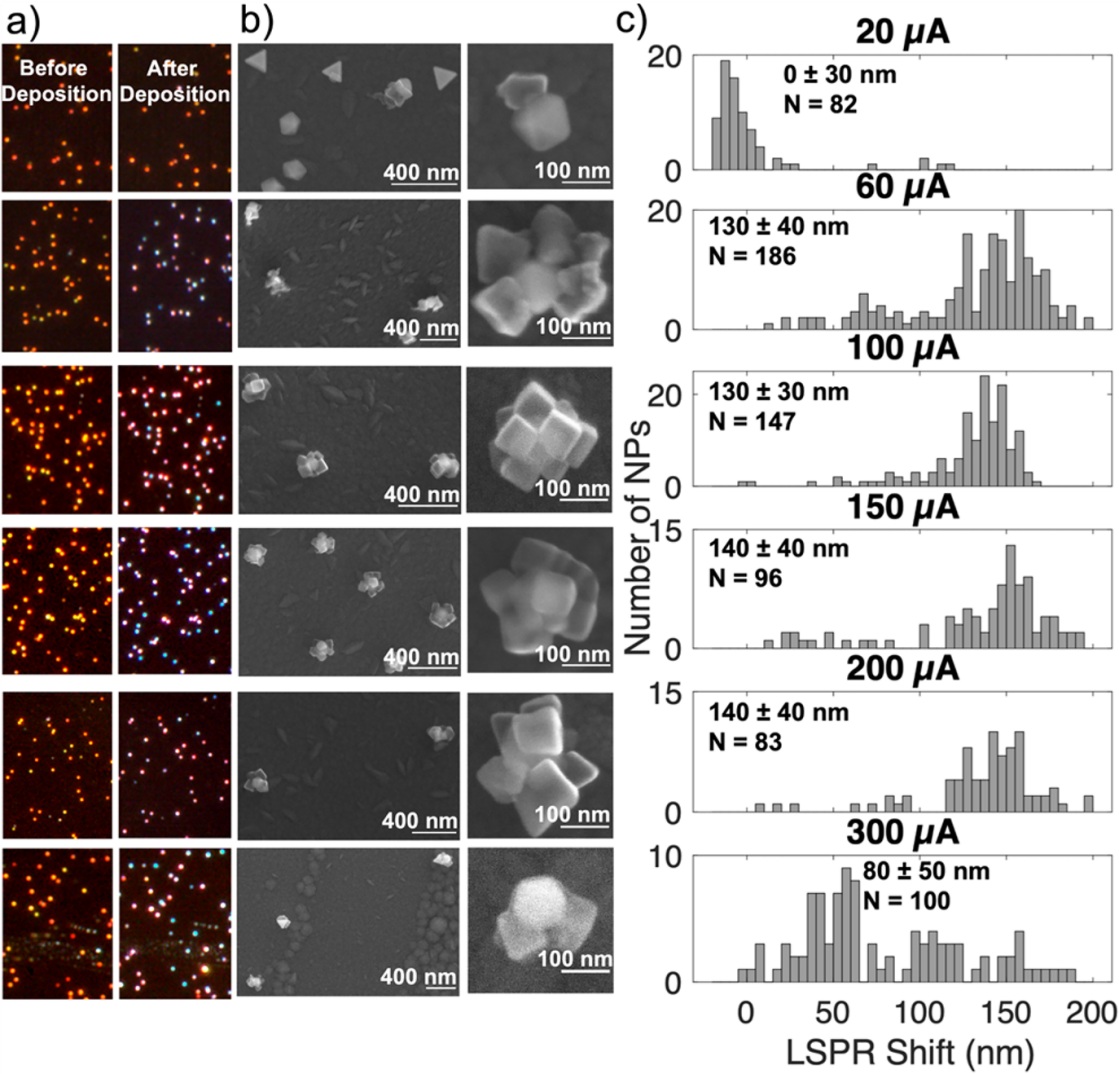
Effect of current on Cu electrodeposition on Au NPs, at a fixed total charge transfer of 0.48 mC. (a) Dark field optical scattering images from the same region before (left) and after (right) deposition. (b) SE SEM images of representative NPs after deposition and (c) LSPR shifts after electrodeposition, with the average, standard deviation, and number of NPs (*N*) reported on each histogram.

**Figure 4:**
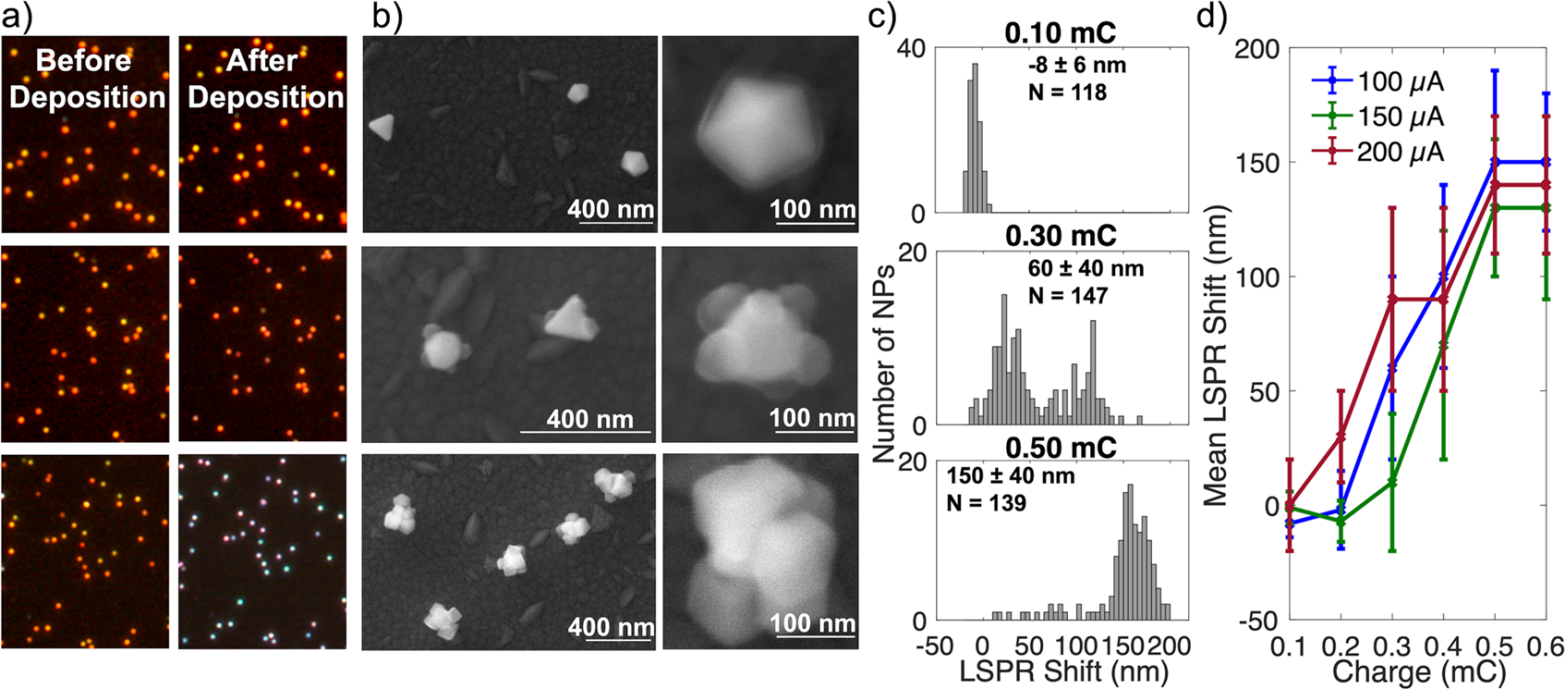
Effect of total charge transfer on Cu electrodeposition on Au NPs. (a) Dark field optical scattering images from the same region before (left) and after (right) deposition, (b) SE SEM images of representative particles after deposition, (c) LSPR shifts after electrodeposition for total charge transfers of 0.10, 0.30, and 0.50 mC at a fixed current of 100 μA with the average, standard deviation, and number of NPs (*N*) reported on each histogram, and (d) average LSPR shifts and standard deviation as a function of both charge transfer and deposition current, from the data presented in (c) and Figures S15–17.

The magnitude of the LSPR shift, 100 nm for the NP in Figure 2, with more statistics shown in Figures 3 and 4, suggests a Cu shell but is not inconsistent with a Cu/Cu_2_O shell depending on its geometry. The morphology of the rough shell of crystallites resides in between a smooth conformal shell and a decoration by sharply faceted cubes. We have thus simulated, using DDSCAT,^37^ the optical response in these two extremes for the two possible compositions of the shell (given XPS and XRD), Cu and Cu_2_O. Numerical results for the Au decahedron and the Au decahedron with Cu or Cu_2_O cubes decorating the exposed tips (Figures 2e and S5) reveals a rather large redshift of the azimuthal dipole upon decoration, consistent with the experimental results (Figures 2, 3, and 4). In this cube-on-decahedron geometry, Cu and Cu_2_O yield different intensities but comparable azimuthal dipole wavelengths. The position of the azimuthal dipolar resonance is significantly different, however, when Cu and Cu_2_O are spread around the decahedron as a conformal shell, as shown in Figures 2 and S5. In this case, as in the case for a sphere (Figure S6), the magnitude of the shift expected upon deposition is smaller for Cu and matches our experimental data. The redshift for a conformal Cu shell (∼80 nm) is smaller than that for the decoration by Cu cubes (∼270 nm). The experimental LSPR shift data is thus not inconsistent with metallic Cu deposition,^57^ considering the morphology obtained experimentally is somewhere in between a shell and a cube-decorated structure.^57^

### Optimization of Deposition Conditions

The electrodeposition setup used allowed for tuning the substrate NPs’ type and density, the deposition solution, and the electron flow. These parameters were explored, and the results are briefly summarized below, with additional data and figures in the Supporting Information.

Increasing or lowering the density of Au NPs drop cast on the ITO working electrode, from the standard 680 ± 30 NPs/mm^2^ up to 2600 ± 100 NPs/mm^2^ and down to 270 ± 30 NPs/mm^2^, modified as expected the amount of Cu deposited per Au NP. The density of Cu nucleation on the Au remained roughly constant for all densities, but less growth was observed on higher density substrates (Figure S7). Analogously, the [CuSO_4_] in solution impacted nucleation (Figure S8). At the low concentration of 0.040 mM CuSO_4_, no shell-like structures on Au were observed or Cu detected in SEM-EDS (Figure S9), and the chronopotentiometry signal (Figure S10c) suggests depletion of Cu at the electrode; at high concentration (25 mM CuSO_4_) the deposition became highly heterogeneous. Meanwhile, at intermediate concentrations (0.20 to 5.0 mM CuSO_4_), the Cu morphology on Au changed slightly, with, on average, more Cu nuclei at low concentration, but importantly, at 5.0 mM Cu nucleation on the ITO substrate was minimized. Thus, this concentration was chosen for further experiments. This observation is consistent with the findings of Grujicic and Pesic^48^ who observed an increase in nucleus size and decrease in nuclei population with an increase in [CuSO_4_]. Decreasing the pH of the solution from 7 to 2 did not improve the shell’s homogeneity, yielding fewer larger Cu crystals at pH 4 and no deposition at pH 2 (Figure S11). pH effects on the morphology of electrodeposited Cu have previously been observed;^58^ however, this published work was on Cu electrodes from pH 0.5 to 4.5 with much higher [CuSO_4_] (∼120 mM), making direct comparison difficult. For simplicity and because it produced deposition mainly on the NPs, a neutral pH was used for the remainder of the studies.

We investigated the effect of sweeping the potential downward during deposition, which resulted in irregular Cu structures with poorer deposition control than the fixed current approach (Figure S12). However, potential sweeps suggest that no underpotential deposition (UPD) occurs: indeed, noticeable LSPR shifts, signature of deposition onset, start from −150 to −180 mV rather than the >−140 mV expected for UPD. This lack of UPD contrasts, because of the composition of the working electrode, with the behavior of Cu on Ag NPs reported in ref (32). This observation is further supported by the lack of a sharp prepeak in our cyclic voltammograms (CV, Figure S13b), another indicator of UPD.

### Effect of Deposition Current

In controlled current electrodeposition, an increase in current is enabled by a decrease in potential (more negative potential, Figure S10b). This change in potential changes the deposition overpotential and can be used to manipulate nucleation density.^59^ We investigated this effect on the electrodeposition of Cu on Au by varying the deposition current between 20 and 300 μA while keeping the total charge transfer constant at 0.48 mC. As expected, at the very high current of 300 μA (and potential around −250 mV, Figure S10b), nucleation readily occurred, and many Cu NPs were formed on the ITO surface as well as on the Au NPs, leading to less Cu per Au NP and smaller LSPR shifts (Figure 3). Conversely, few rather large crystallites were deposited at 20 μA (−130 mV), with most Au NPs not covered and hence their optical response unaffected. At moderate currents, smaller crystallites were obtained as shown in Figure S14 for 150 μA. Also at moderate currents, between 60–200 μA, comparable shifts were obtained (Figure 3), as follows: 130 ± 40 nm at 60 μA (*N* = 186), 130 ± 30 nm at 100 μA (*N* = 147), 140 ± 40 nm at 150 μA (*N* = 96), and 140 ± 40 nm at 200 μA (*N* = 83). Of those, the 60 μA depositions had the poorest uniformity as observed in SEM images and the LSPR shift distribution, such that we concluded that the 100–200 μA range was optimal.

### Effect of Total Charge Transfer

The number of electrons consumed at the working electrode correlates with how much Cu is reduced; therefore, controlling the total charge transferred provides a way to manipulate Cu electrodeposition. We varied the charge transfer from 0.10 to 0.60 mC at constant currents: results for 100 μA are shown in Figures 4 and S15, while those for 150 and 200 μA are shown in Figures 4d and S16–17. As expected, an increase in total charge transfer led to an increase in Cu deposition and in the magnitude of the associated LSPR redshift. This control over the extent of deposition offers an insight into the growth mechanism by providing snapshots along the growth path. At 0.1 mC, there was no noticeable Cu deposition on the Au NPs, as confirmed by SEM-EDS (Figure S18). Then, in the early stages, i.e., at small total charge transfers such as 0.20 or 0.30 mC (Figures S15–17), multiple Cu nuclei appeared on the Au NPs. With an increase in total charge transfer, growth of pre-existing Cu was favored over the formation of many additional crystallites, suggesting a nucleation and then growth mechanism as described by Guo et al.^60^ Unfortunately, we do not have images of the same NPs after several depositions due to the need to avoid beam-induced effects; however, we did not observe noticeably more Cu crystallites per NP in large compared to small total charge transfers. Instead of further nucleation, increasing the total charge transfer appeared to grow the crystallites until they became adjacent to each other and formed a lumpy shell, in a uniform manner from NP to NP.

### Time Resolved Studies

**Figure 5:**
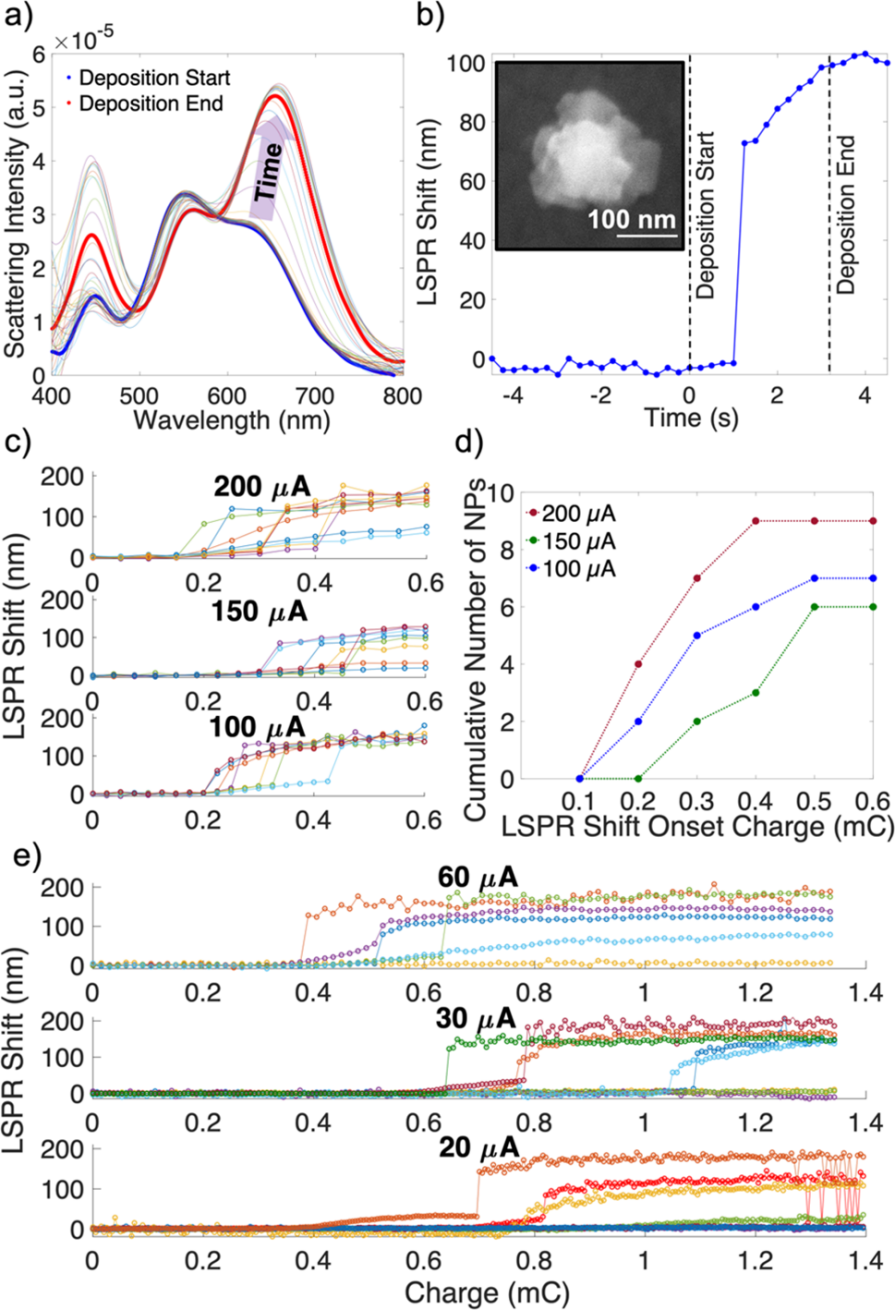
Single particle tracking during electrodeposition. (a) Spectral evolution with time during the electrodeposition of Cu on a single Au NP, (b) LSPR shift with time for the data shown in (a), with an SEM image of the same NP in the inset, (c) LSPR shift per NP as a function of charge for depositions at 200, 150, and 100 μA, (d) summary of the LSPR onset charge for 200, 150, and 100 μA, and (e) LSPR shift per NP as a function of charge for 60, 30, and 20 μA, showing a greater heterogeneity in the onset charge and more delayed LSPR shifting onsets than those in (c).

The rapid nucleation suggested by the rough NP morphology is supported by time-resolved optical studies. Here, we acquired the scattering spectra of single particles immersed in the electrolyte solution during deposition and present them as a function of total charge transfer for various currents (Figure 5). Shifts in the LSPR tracked the formation of Cu crystallites on Au NPs; for most NPs no shift was observed at first, followed by a rapid redshift and increase in intensity, after which a slow increase or plateau occurred. The onset of this LSPR shift varied with current. At high current, nucleation occurred more rapidly both in time and in amount of total charge transfer (Figure 5), as expected from a higher nucleation driving force. At lower currents (*e.g.*, 20–60 μA) the nucleation was slower and its onset more variable from NP to NP, leading to the less homogeneous deposition observed in SEM images (Figure 3b).

### Deposition on Plasma Treated Au NPs

**Figure 6:**
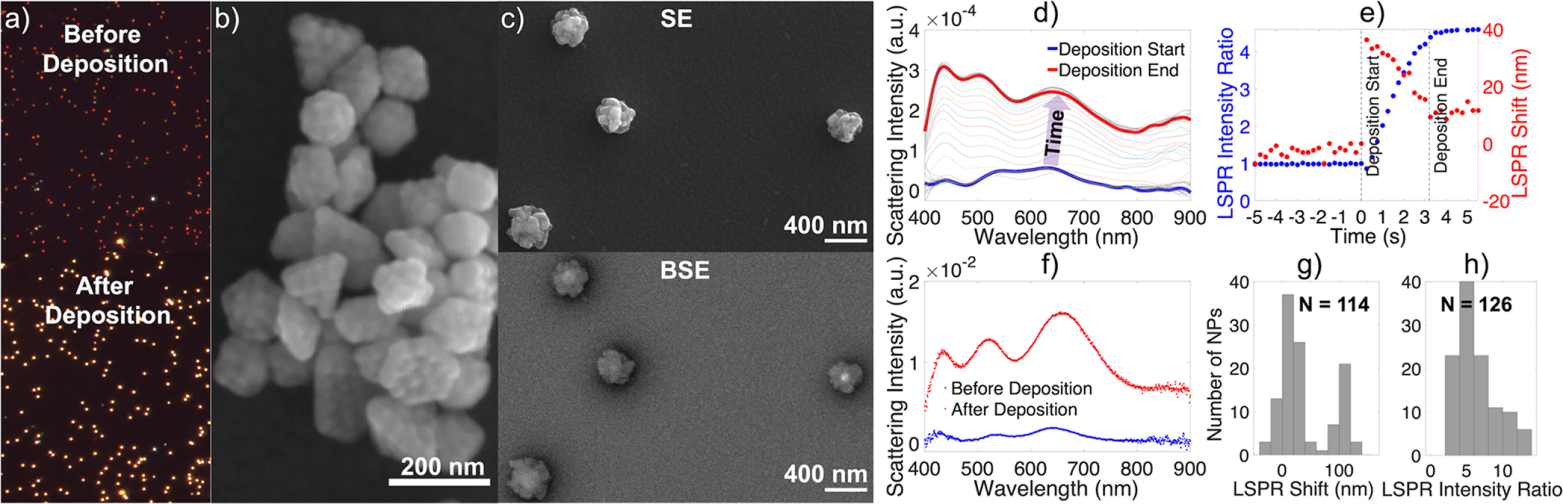
Electrodeposition on plasma treated Au NPs. (a) Dark field optical scattering images from the same region before (top) and after (bottom) deposition with a current of 150 μA and a total charge transfer of 0.48 mC, (b) SE SEM images of Au NPs after a 5 min, 20 W Ar/O_2_ plasma treatment, showing pitting, (c) SE (top) and BSE (bottom) SEM images of pitted Au NPs after electrodeposition of Cu, (d) representative single NP LSPR spectra obtained in solution during deposition, (e) LSPR shift and intensity ratio as a function of time for the NP in (d), (f) representative single NP spectrum acquired in air before and after deposition, (g) LSPR shifts after electrodeposition, and (h) scattering intensity increase reported as the ratio of the final over the initial peak intensity.

Treating Au NPs with O_2_/Ar plasma (5 min at 20 W) resulted in a roughening of the Au NP surface, along the lines of what was observed for N_2_ plasma-generated triangular pitting patterns.^61^ This increased the surface roughness significantly, leading to more uniform and much thicker Cu shells. For deposition at a current of 150 μA and a total charge transfer of 0.48 mC (Figure 6), the shells obtained were ∼110 nm thick, much larger than the 45 nm obtained on nonplasma treated NPs (although the latter have lumpier shells). We hypothesize that these thicker, more uniform shells are due to the increase in nucleation sites on the roughened Au NPs, leading to a larger number of smaller, more evenly distributed Cu crystallites. This hypothesis is further supported by the disappearance of the preferential nucleation at the tips of NPs observed previously; indeed, with so many nucleation points and high curvature areas on the surface, the tips lost their uniqueness.

Changes in morphology were accompanied by changes in optical properties: the plasma treatment resulted in a slight blueshift due mainly to the loss of PVP (Figure S19); pitting was shown to affect only slightly the LSP energy.^61^ Electrodeposition then led to a redshift and a significant increase in scattering intensity (Figure 6). The magnitude of the deposition-induced shift was smaller than expected for the creation of such a thick shell. This effect can, in small part, be attributed to the blue shift caused by infilling the pitted structure. The dominant cause, however, is the stark morphology difference between the Cu/Au structures obtained from plasma-treated Au NPs and PVP-capped Au NPs: the latter have chunky cubes grown at their highly sensitive tips, leading to significant redshift, while Cu deposition on the latter occurs also on facets and forms a looser shell. PVP removal by NaBH_4_ cleaning blueshifted the spectra in a similar fashion as plasma cleaning (Figure S20) but did not produce Au pitting nor affect the resulting Cu morphology, which remained dominated by cubes on tips (Figure S2).

### Oxidation of Cu on Au NPs Postdeposition

**Figure 7:**
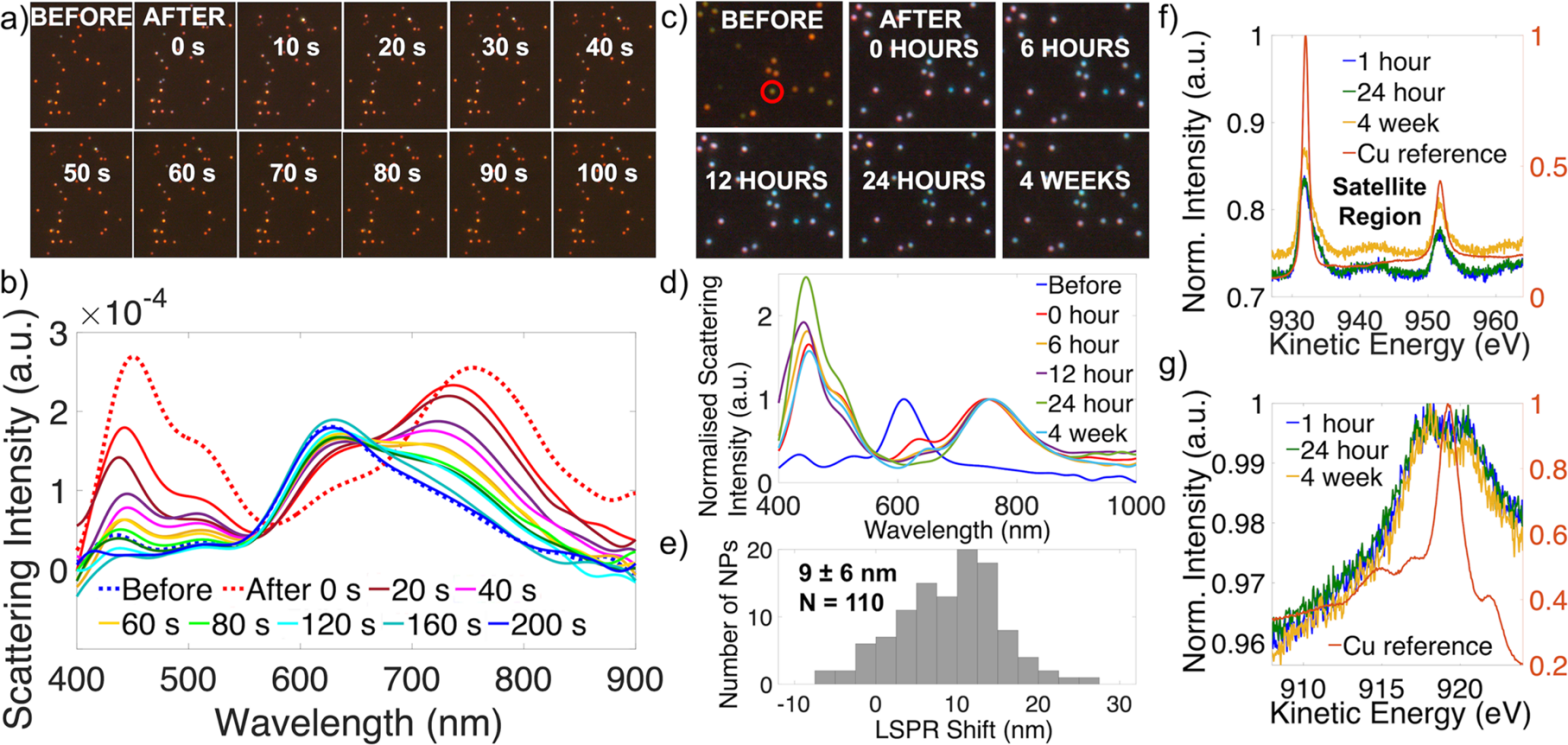
Stability of Au with electrodeposited Cu NPs in solution and in air after deposition. (a) Dark field optical scattering images in solution before (top left) and at time intervals after deposition, (b) *in situ* single particle scattering spectra in aqueous solution tracking the LSPR evolution before and at time intervals after deposition, (c) dark field optical scattering images in air at different times postdeposition, (d) normalized scattering spectra for a single NP (red circle in (c)) in air before and after deposition, and (e) histogram of the LSPR shift between 0 h and 4 weeks in air postdeposition for 110 NPs. (f and g) XPS 2p and LMM Auger spectra after 24 h and 4 weeks in air postdeposition, where the orange scale corresponds to the normalized intensity of the Cu reference.

All measurements, except those during deposition, shown so far were performed in air to avoid oxidation in aqueous solution, which was fast and significant. In Figure 7, we show the extent of oxidation in the electrolyte solution in contrast with the stability of the NPs in air. There is of course a shift between air and solution, as shown in Figure S21, which affects the starting LSPR position. Then, in solution (Figure 7a,b), both dark field optical scattering spectra and images significantly changed immediately after deposition and returned to their initial, predeposition state after as little as 200 s when no potential was applied. This occurred by the oxidative dissolution of metallic Cu back to its ions and was accelerated by the presence of sulfide ions^62^ in the electrolyte. However, when the NPs were rinsed and dried after deposition and then stored in air for up to 4 weeks, only slight changes were seen in both dark field optical scattering spectra and images, with a small redshift of 9 ± 6 nm (*N* = 110) observed after 4 weeks (Figure 7c–e). This shift indicates further oxidation of the deposited material, ruling out that it was in a fully oxidized state initially. Further, XPS and Auger rule out CuO through the process (no strong satellite peak emerged at 935–945 eV in the Cu 2p spectrum, Figure 7f), such that we conclude that the LSPR shift is due to the oxidation of deposited Cu to Cu_2_O. This is supported by the observed small change in the height of the peak attributable to metallic Cu (right-hand side, Figure 7g) with time and is consistent with the 12 nm LSPR shift observed by Siampour et al. upon oxidation of Cu to Cu_2_O on Au NPs.^47^

## Conclusion

We achieved bimetallic Cu on Au NPs with controllable morphology and optical properties using fixed current electrodeposition. Using this approach, multilobed structures were synthesized with good homogeneity across samples through optimization of deposition current, total charge transfer, and metal ion concentration. The plasma treatment of Au NPs before deposition resulted in larger bimetallic Cu on Au NPs with a more uniform shape.

Single particle spectra and their LSPR shifts were measured during the deposition process, giving an insight into the deposition kinetics and informing the better selection of experimental parameters for homogeneous deposition. In air, Cu on Au NPs were found to be stable for at least 4 weeks, while Cu rapidly oxidized in aqueous solution, as revealed by single particle optical spectroscopy. Overall, this technique provides a toolbox for the electrodeposition of bimetallic plasmonic NPs and opens the door for the future production and utilization of different combinations of core and electrodeposited metal.

## Supporting Information Available

HAADF STEM and STEM-EDS line scans, SEM-EDS spectra, optical and electron microscopy results for experiments where Au NP density, CuSO_4_ concentration, pH, total charge transfer illumination, and surface conditions are modified, potential sweep electrodeposition, electrochemical curves, control experiments in the absence of Cu, and additional numerical results (PDF)
