# Supplementary material for: Synthesis of Controllable Cu Shells on Au Nanoparticles with Electrodeposition: A Systematic in Situ Single Particle Study
Source: J Phys Chem C Nanomater Interfaces. 2023 Mar 1;127(10):5044–53. doi: 10.1021/acs.jpcc.2c08910 (PMC10026066; doi:10.1021/acs.jpcc.2c08910)
Supplement: Supplementary file 1 — jp2c08910_si_001.pdf [file jp2c08910_si_001.pdf]

Supporting Information for

Synthesis of Controllable Cu Shells on Au Nanoparticles with Electrodeposition: A Systematic *In-situ* Single Particle Study

Mohsen Elabbadi, [1,2] Christina Boukouvala,[1,2] Elizabeth R. Hopper,[1,2,3] Jérémie Asselin,[1,2] Emilie Ringe[1,2]*

1. Department of Materials Science and Metallurgy, University of Cambridge, 27 Charles Babbage Road, Cambridge, United Kingdom, CB3 0FS
2. Department of Earth Sciences, University of Cambridge, Downing Street, Cambridge, United Kingdom, CB2 3EQ
3. Department of Chemical Engineering and Biotechnology, University of Cambridge, West Cambridge Site, Philippa Fawcett Drive, Cambridge, United Kingdom, CB3 0AS

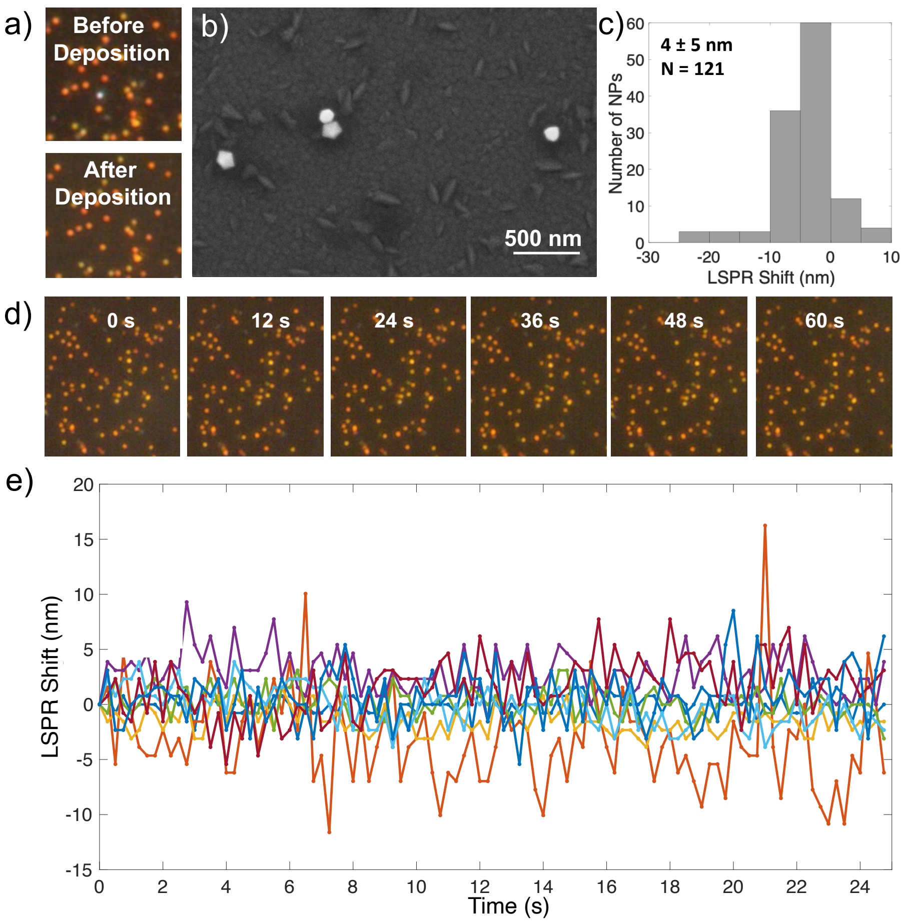

Figure S1. Effect of applying a current in the absence of $CuSO_4$ on deposition. a) Dark field optical scattering images from the same region before (top) and after (bottom) deposition, b) SE SEM image of representative NPs after deposition, and c) histogram of LSPR shift at 150 μA for 3.2 s. d) Dark field optical scattering images from the same region at different deposition times, and e) LSPR shift of different NPs as a function of time at a deposition current of 60 μA.

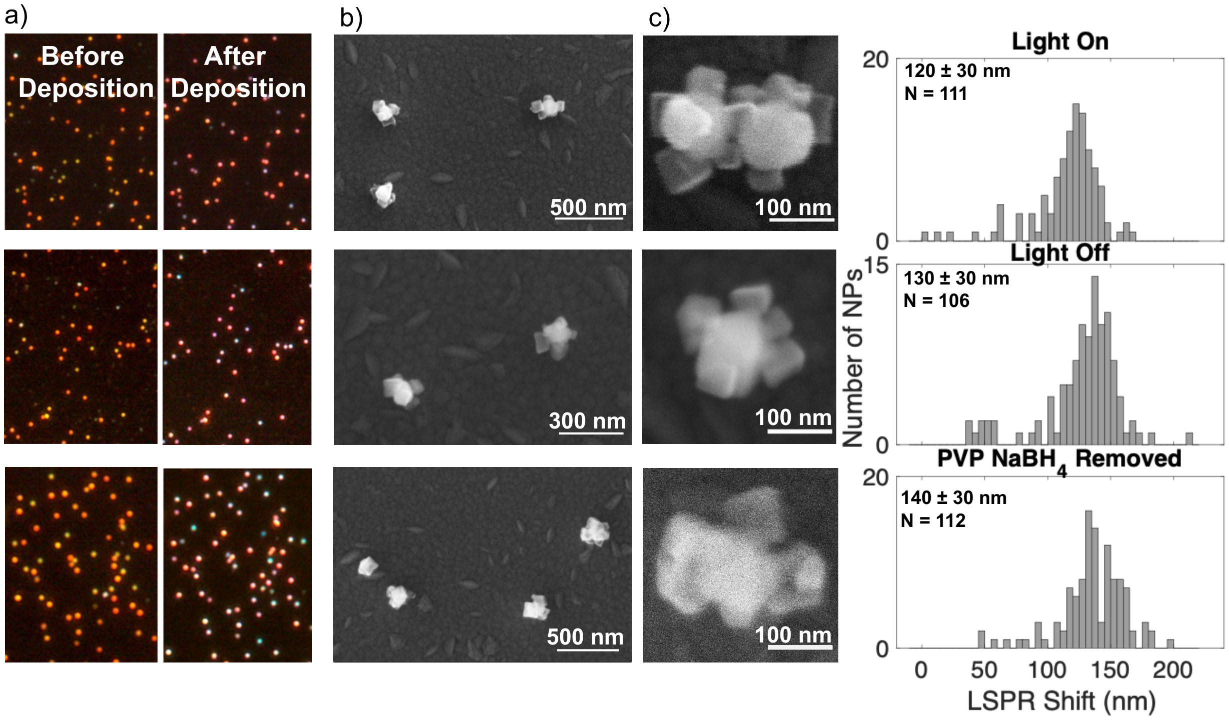

Figure S2. Effect of illumination and PVP removal by $NaBH_4$ on Cu on Au deposition at a current of 150 μA for 3.2 s (total charge transfer of 0.48 mC). a) Dark field optical scattering images from the same region before (left) and after (right) deposition, b) SE SEM images of representative NPs after deposition, and c) LSPR shifts after electrodeposition with the average, standard deviation and number of NPs (N) reported on each histogram.

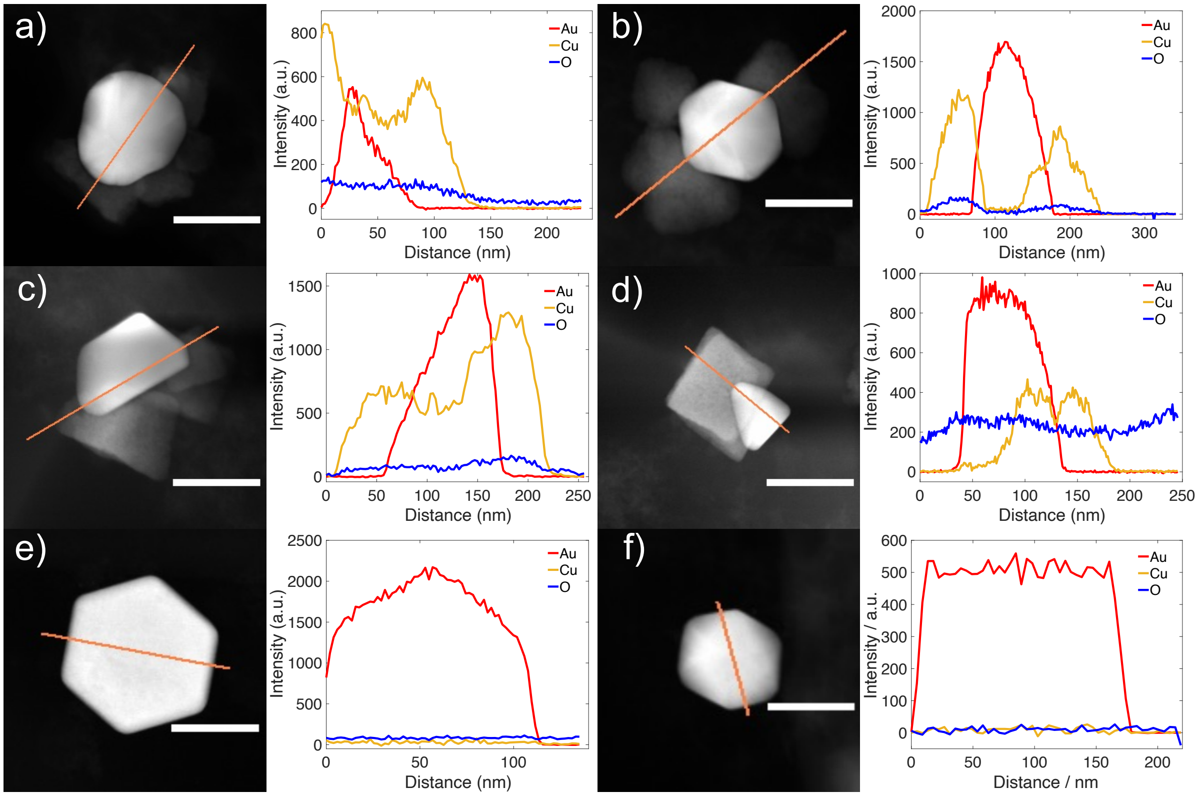

Figure S3. High angle annular dark field scanning transmission electron microscopy (HAADF-STEM) images and STEM energy dispersive X-ray spectroscopy (STEM-EDS) line scans for different Cu on Au NPs from a deposition at 150 μA for 3.2 s (total charge transfer of 0.48 mC). NPs with no apparent Cu nucleation from the HAADF-STEM images, for instance e) and f), produce no Cu signal in the EDS spectrum. Scale bars, 100 nm.

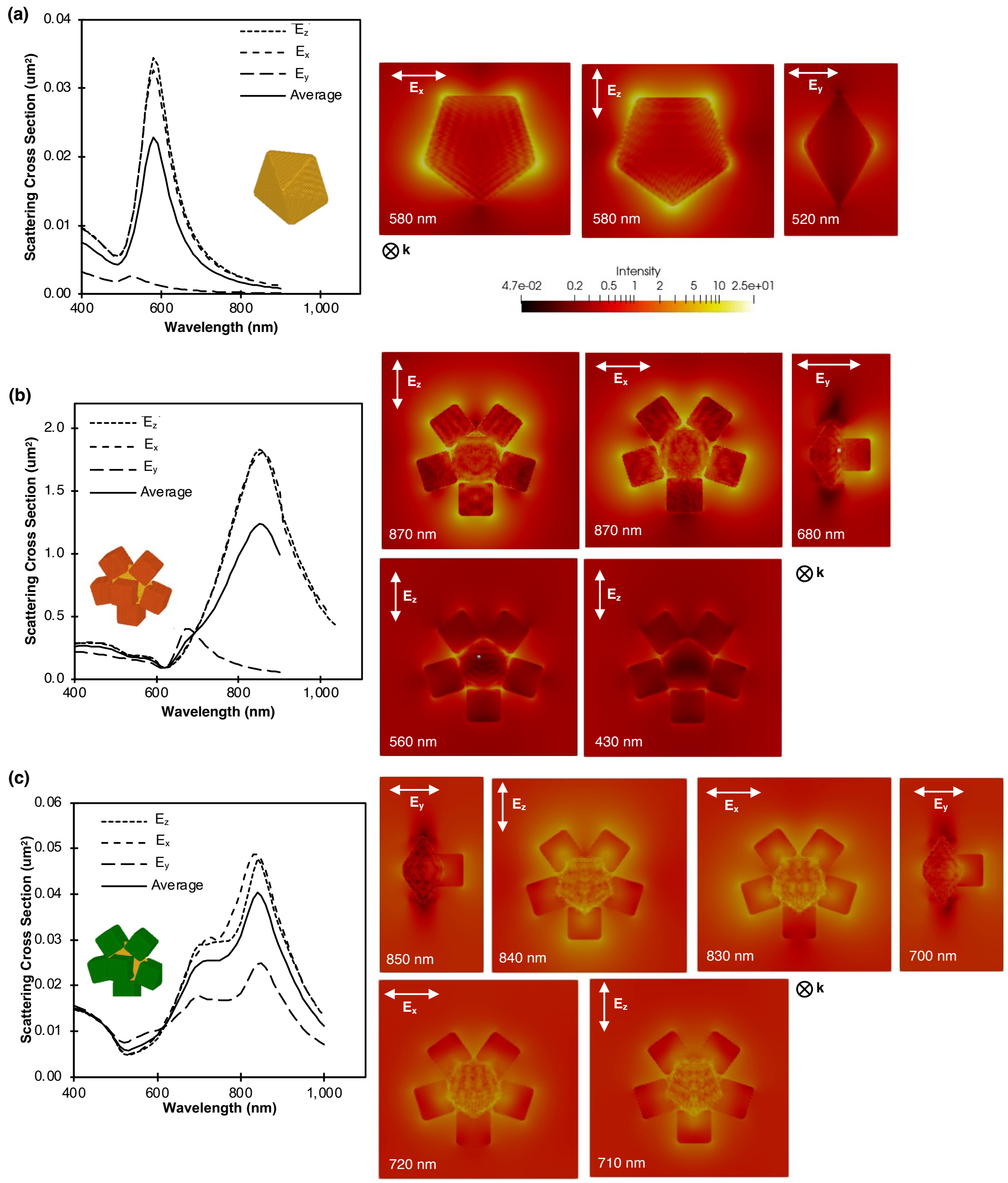

Figure S4. Scattering cross sections for three orthogonal polarizations (dashed lines) and their average (solid lines) along with the field distributions on the most prominent peaks for (a) Au decahedron, (b) Au decahedron decorated with Cu cubes and (c) Au decahedron decorated with Cu_2O cubes. Light propagation (k) directions are denoted with (x) and polarization directions (E) with a double arrow.

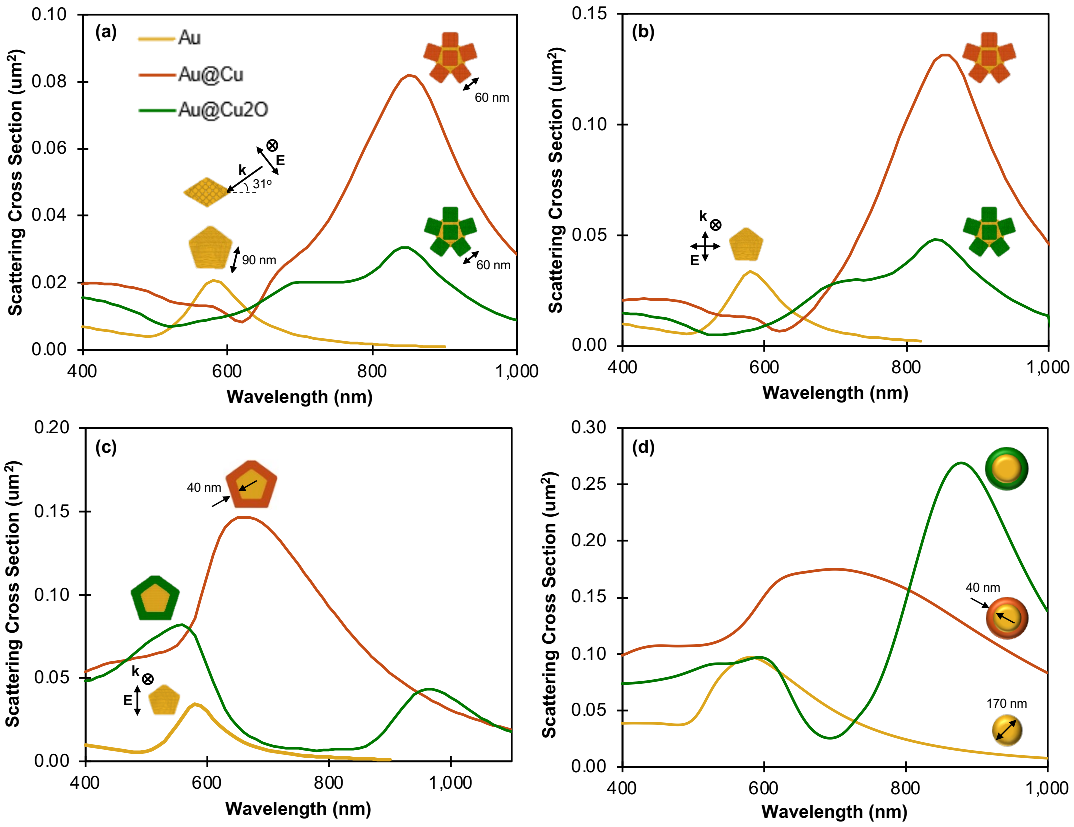

Figure S5. Scattering cross sections for a Au decahedron, Au decahedron decorated with Cu cubes and Au decahedron decorated with Cu_2O cubes for (a) light propagation direction at 31° with respect to the substrate and (b) light propagation direction along the five-fold axis for orthogonal polarizations as indicated in the inset. The Au decahedral core and Cu and Cu_2O cubes have edge lengths of 90 nm and 60 nm respectively. Scattering cross sections for Au, Au@Cu and Au@Cu_2O structures with a 40 nm conformal shell for (c) decahedra and light propagation direction along the five-fold axis and polarization as shown in the inset, and (d) spheres. The Au decahedral core has an edge length of 90 nm, the Au spherical core a diameter of 170 nm and Cu and Cu_2O shells both have a thickness of 40 nm. Yellow, orange and green colours correspond to Au, Cu and Cu_2O respectively. Light propagation (k) is indicated with a single arrow, polarization (E) with a double arrow and both with (x) when out of plane. Calculations for (a), (b) and (c) were obtained via DDSCAT while STRATIFY was used for those in (d).

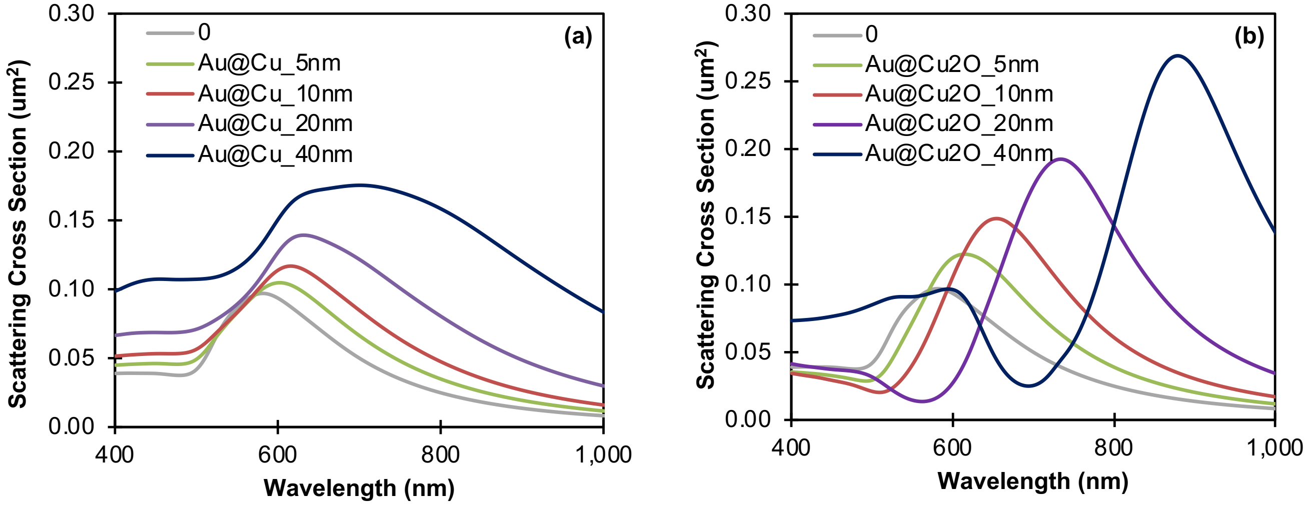

Figure S6. Scattering cross sections for (a) Au@Cu and (b) Au@Cu_2O spheres with varying shell thickness obtained using STRATIFY.

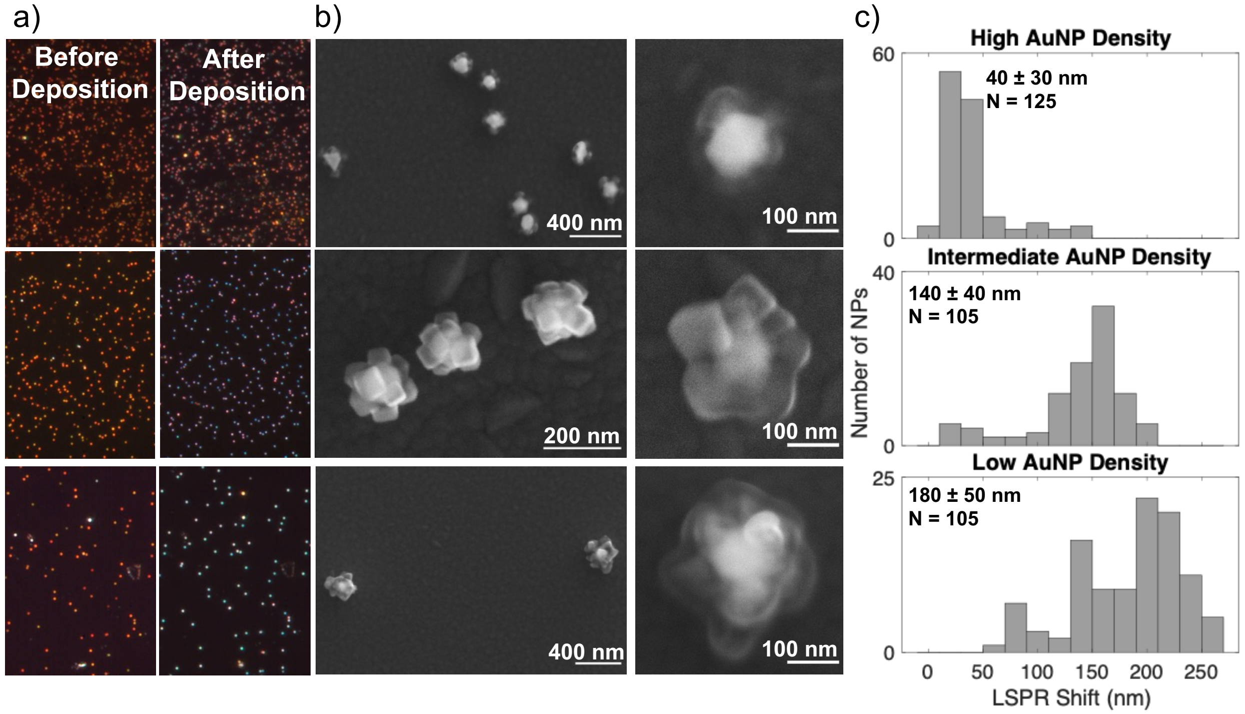

Figure S7. Effect of Au NP density on the electrodeposition of Cu on Au at a current of 150 μA for 3.2 s (total charge transfer of 0.48 mC). Top, middle and bottom rows have 2600 ± 100, 680 ± 30, and 270 ± 30 diffraction limited spots per mm^2 respectively. a) Dark field optical scattering images from the same region before (left) and after (right) deposition, b) SE SEM images of representative NPs after deposition, and c) LSPR shifts after electrodeposition, with the average, standard deviation and number of NPs (N) reported on each histogram.

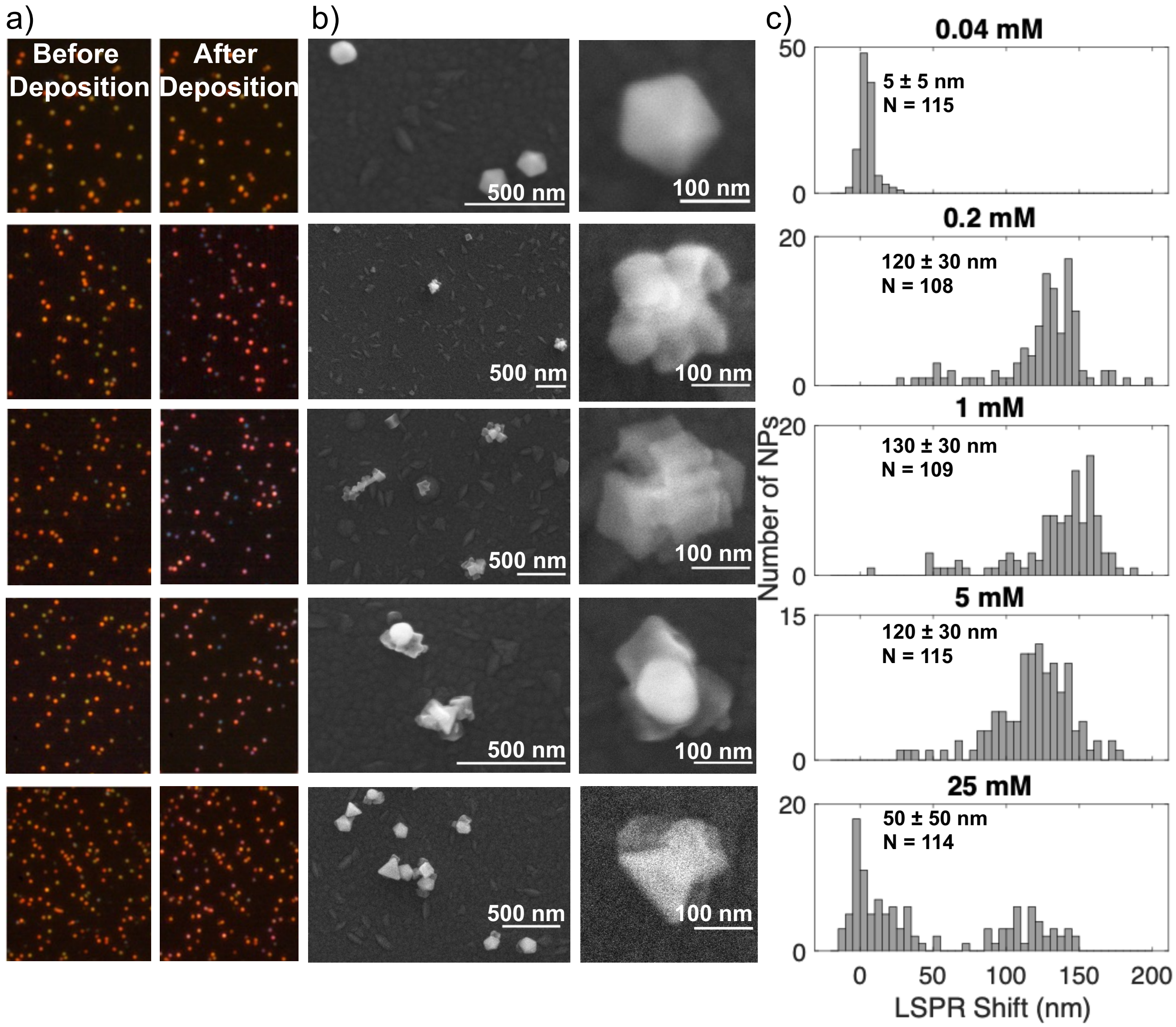

Figure S8. Effect of [$CuSO_4$] concentration on Cu on Au deposition at a current of 150 µA for 3.2 s (total charge transfer of 0.48 mC). From top to bottom, [$CuSO_4$] of 0.040, 0.20, 1.0, 5.0, 25 mM. a) Dark field optical scattering images from the same region before (left) and after (right) deposition, b) SE SEM images of representative NPs after deposition, and c) LSPR shifts after electrodeposition, with the average, standard deviation and number of NPs (N) reported on each histogram.

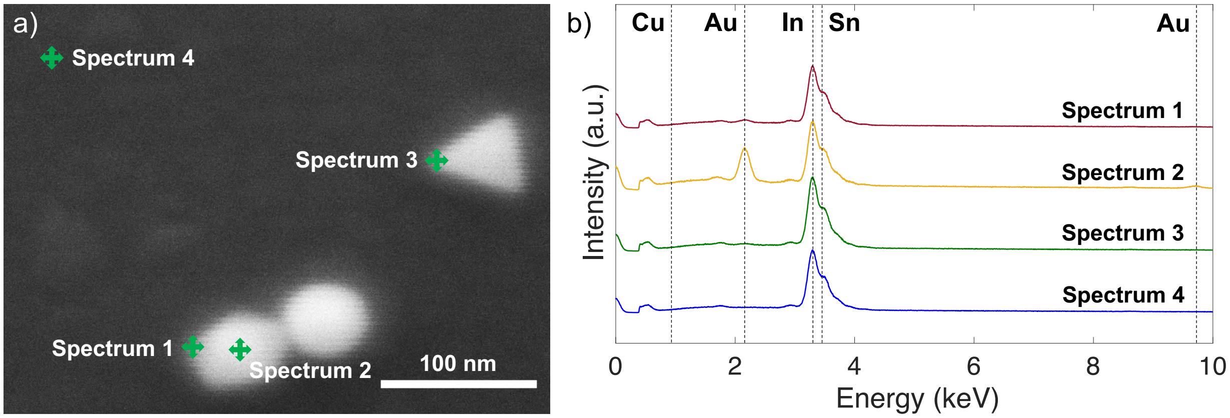

Figure S9. SEM-EDS spectra confirming the absence of Cu. a) SEM image of Au NPs after electrodeposition in 0.04 mM $CuSO_4$, and b) SEM-EDS spectra of regions selected in a.

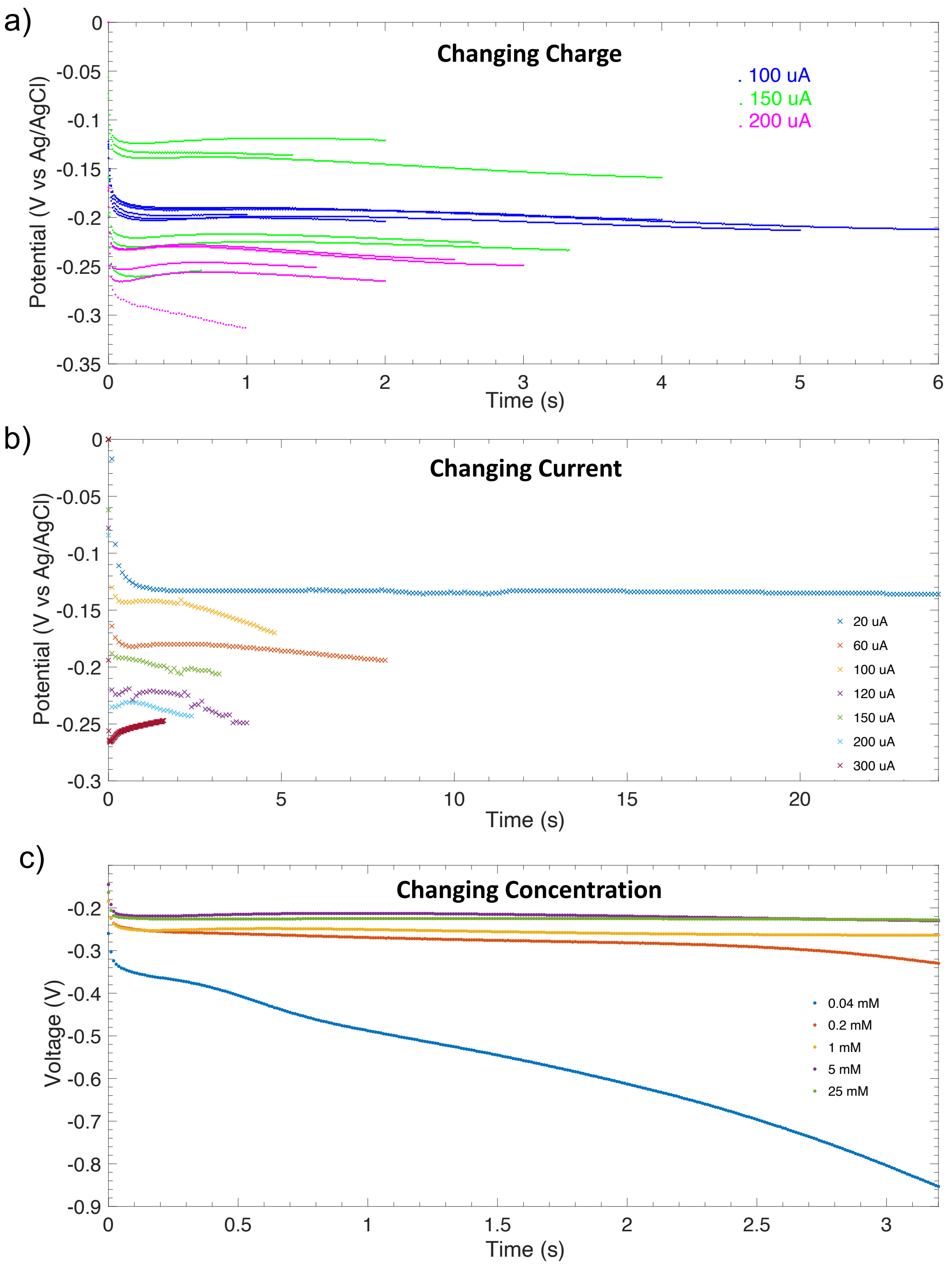

Figure S10. Chronopotentiometry curves for a) changing charge at 100 µA, 150 µA and 200 µA, b) changing current for a constant charge transfer of 0.48 mC, and c) changing [$CuSO_4$] experiments at 150 µA for 0.48 mC.

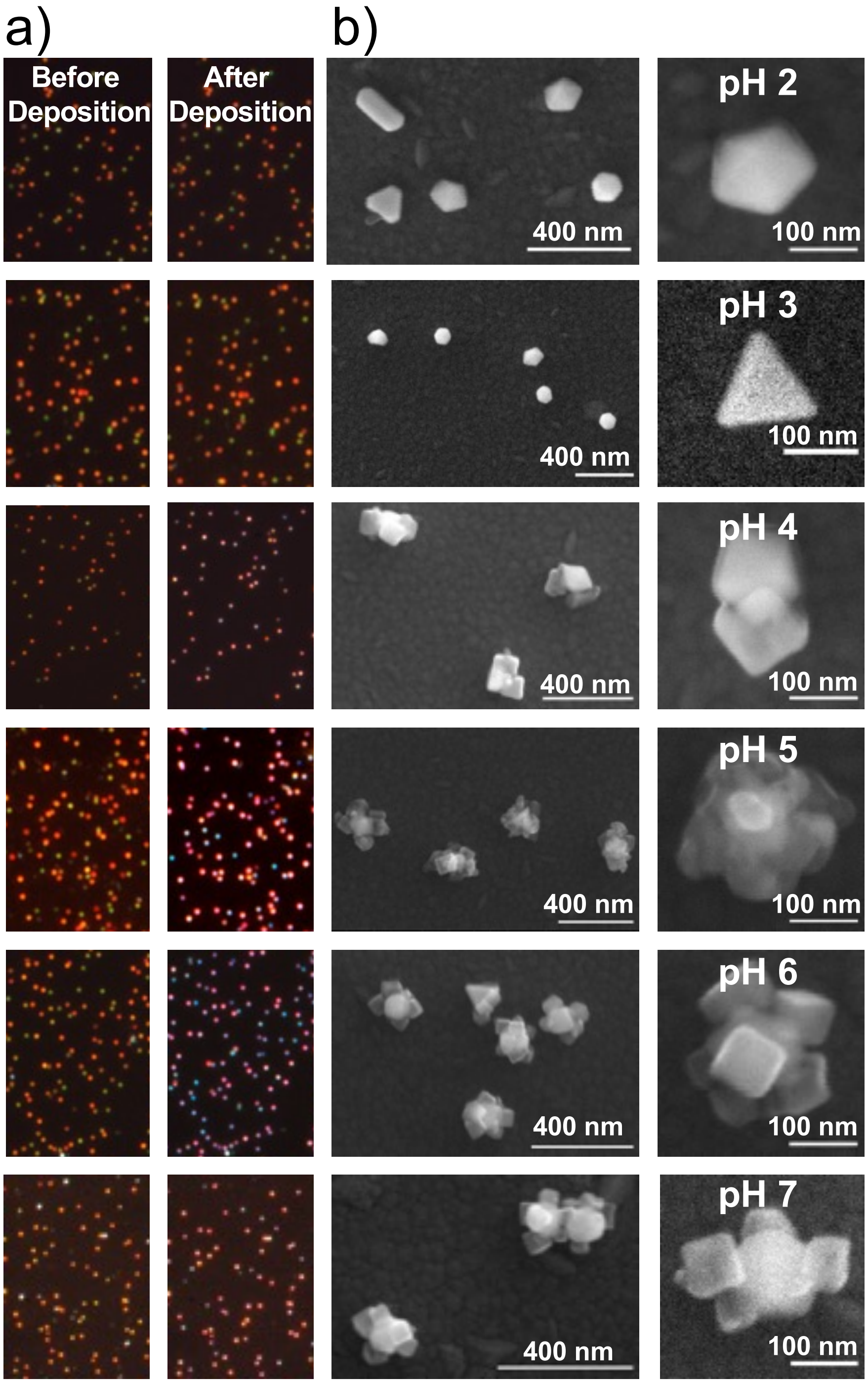

Figure S11. Effect of pH on Cu on Au deposition at a current of 150 μA for 3.2 s. a) Dark field optical scattering images from the same region before (left) and after (right) deposition, and b) SE SEM images of representative NPs after deposition for pH 2 – 7.

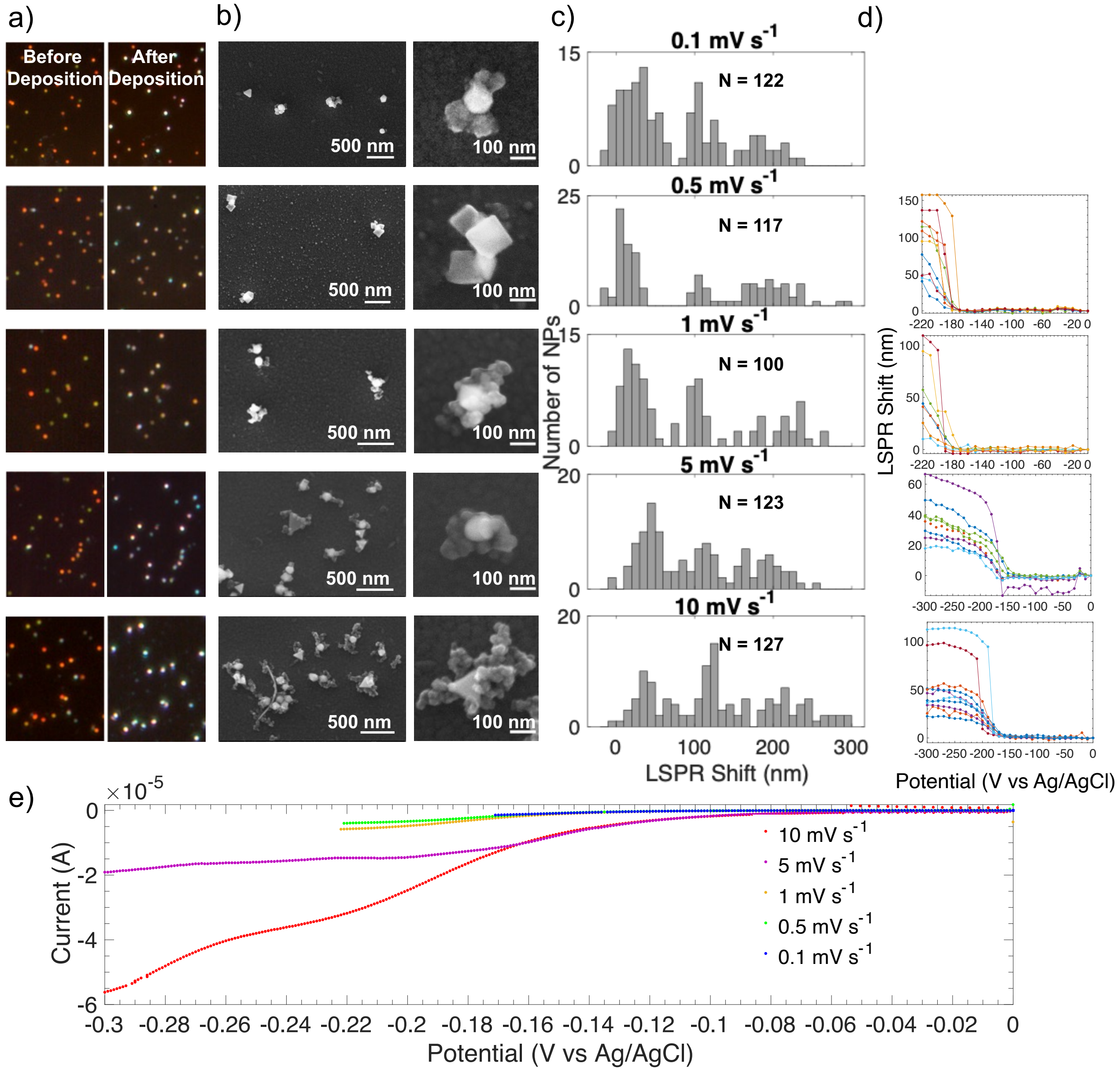

Figure S12. Potential sweep electrodeposition of Cu on Au deposition at different potential scan rates. a) Dark field optical scattering images from the same region before (left) and after (right) deposition, b) SE SEM images of representative NPs after deposition, c) LSPR shifts after electrodeposition, with number of NPs (N) reported on each histogram, d) LSPR shift with potential for single NPs from time-resolved studies, and e) CV curves of deposition.

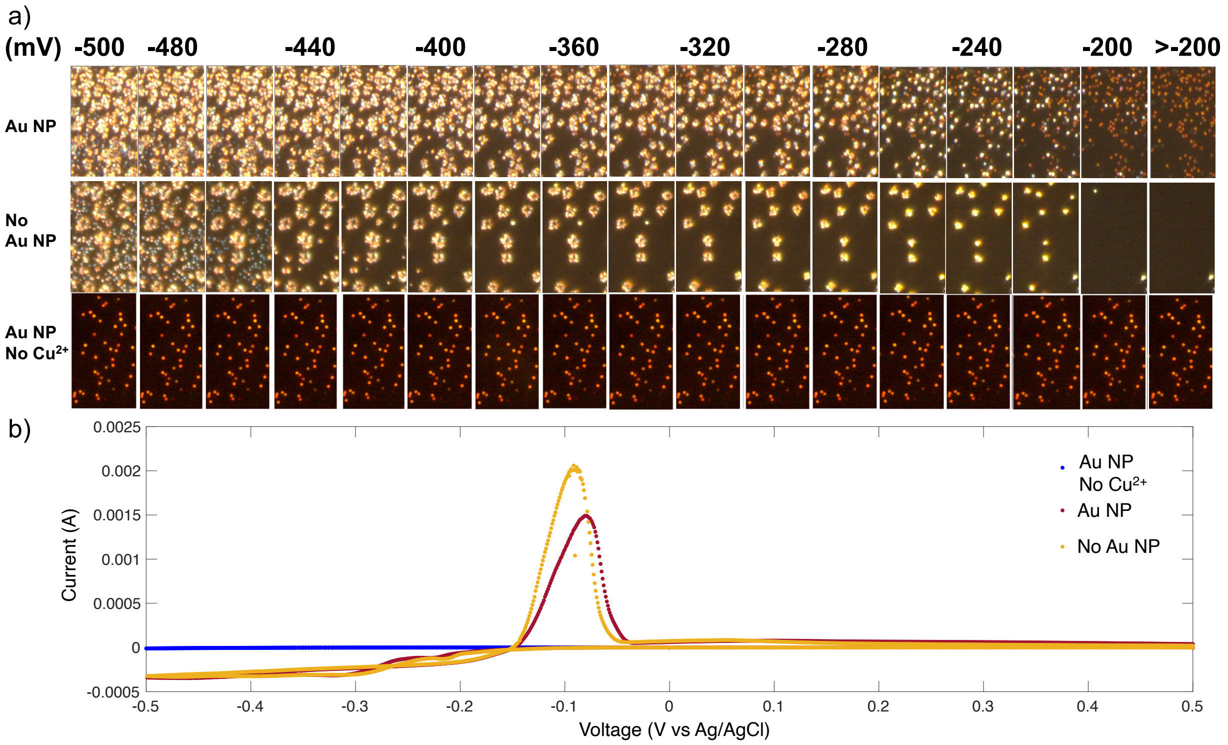

Figure S13. a) Dark field images of deposition of Cu on Au NP and ITO substrate, and in the absence of [$CuSO_4$] on Au NP, at different potentials, b) cyclic voltammograms of 5 mM [$CuSO_4$] and in background electrolyte. Scan rate 1 mV s^{-1}. Onset and peak positions for Au NP and no Au NP very similar, and Cu deposition begins at -200 mV. In the presence of Au NP, Cu nucleation on ITO is seen at a lower potential of -360 mV. In the absence of Cu ions, there no change in dark field scattering observations and no redox peaks are seen.

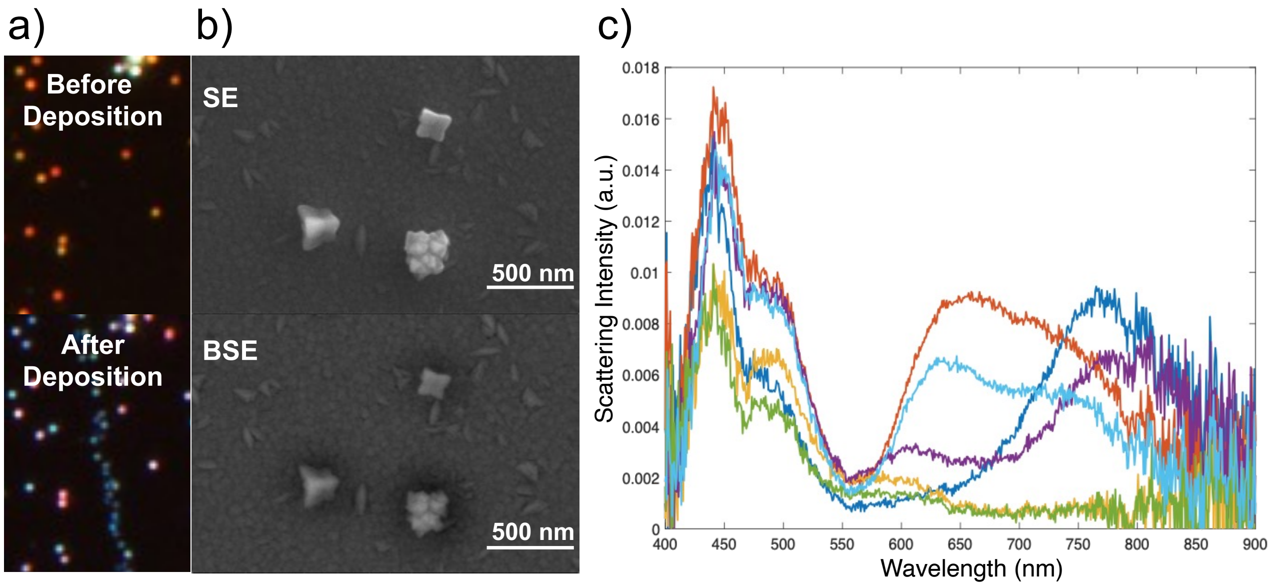

Figure S14. Regions of electrodeposition of Cu on ITO. a) Dark field optical scattering images from the same region before (top) and after (bottom) deposition, b) SE and BSE SEM images of typical morphologies observed, and c) example scattering spectra of Cu NPs deposited on ITO. Deposition at 150 μA for 3.2 s.

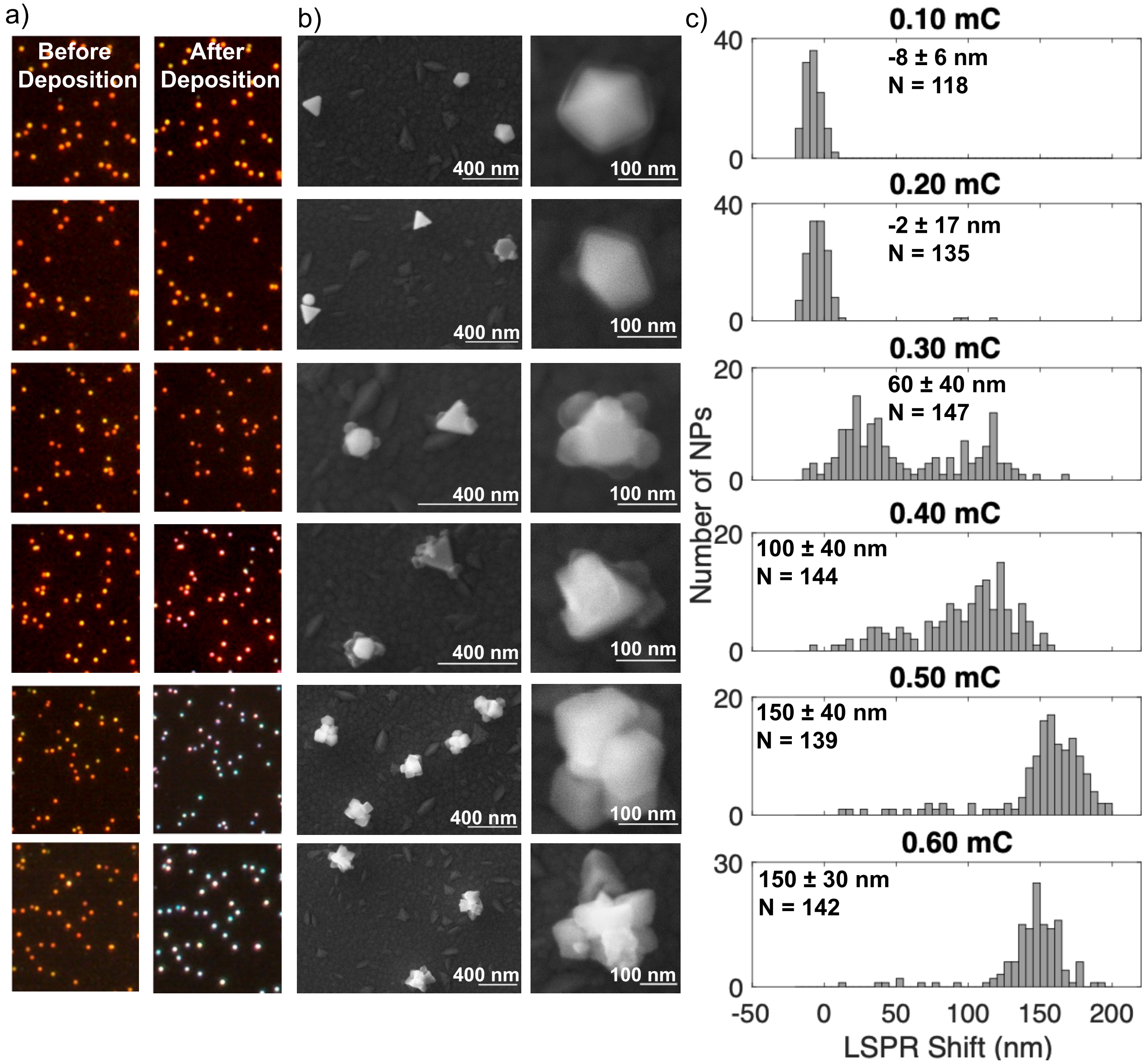

Figure S15. Effect of charge transfer on Cu on Au deposition at a fixed current of 100 μA. a) Dark field optical scattering images from the same region before (left) and after (right) deposition, b) SE SEM images of representative NPs after deposition, and c) LSPR shifts after electrodeposition, with the average, standard deviation and number of NPs (N) reported on each histogram.

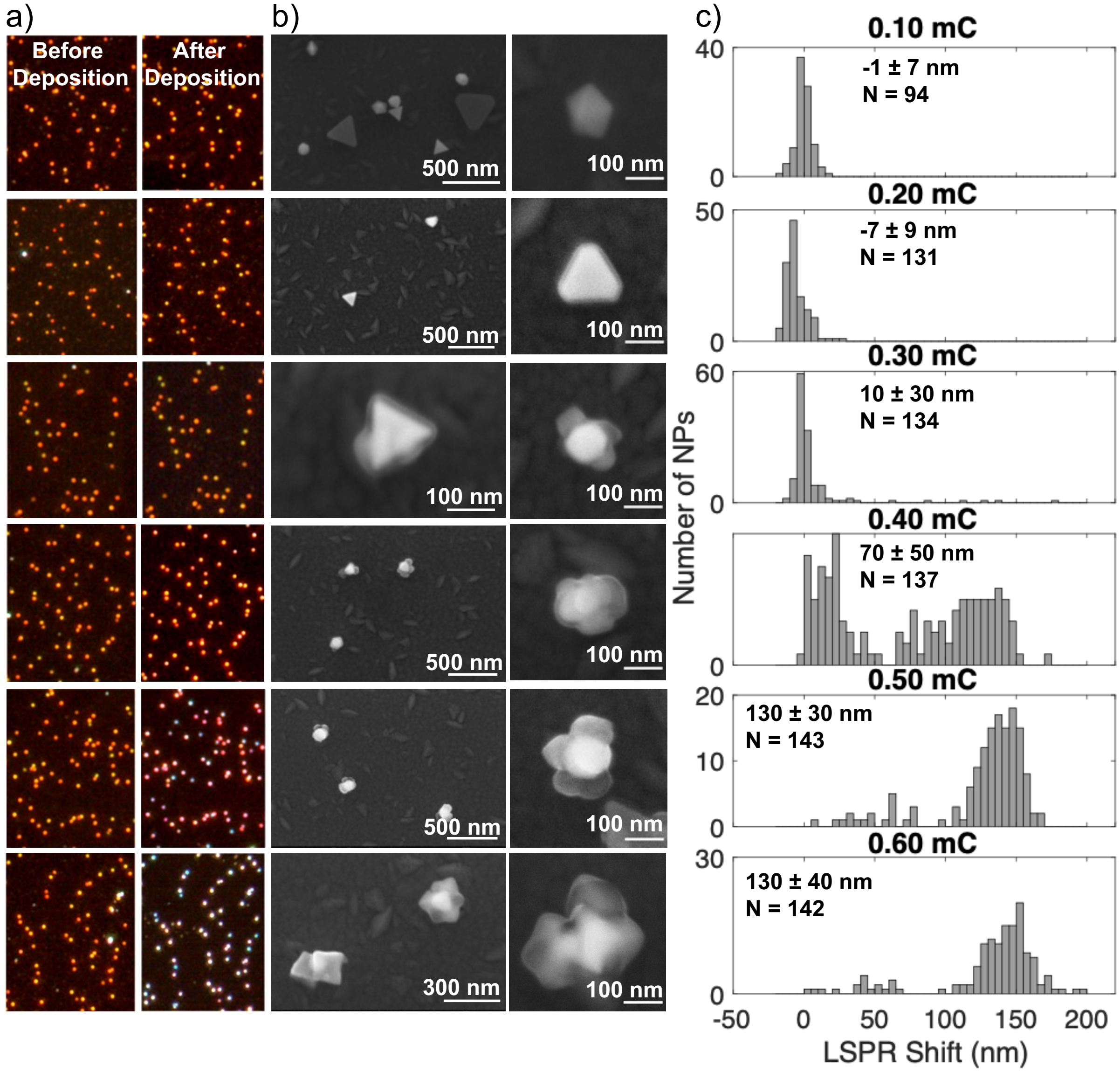

Figure S16. Effect of charge transfer on Cu on Au deposition at a fixed current of 150 μA. a) Dark field optical scattering images from the same region before (left) and after (right) deposition, b) SE SEM images of representative NPs after deposition, and c) LSPR shifts after electrodeposition, with the average, standard deviation and number of NPs (N) reported on each histogram.

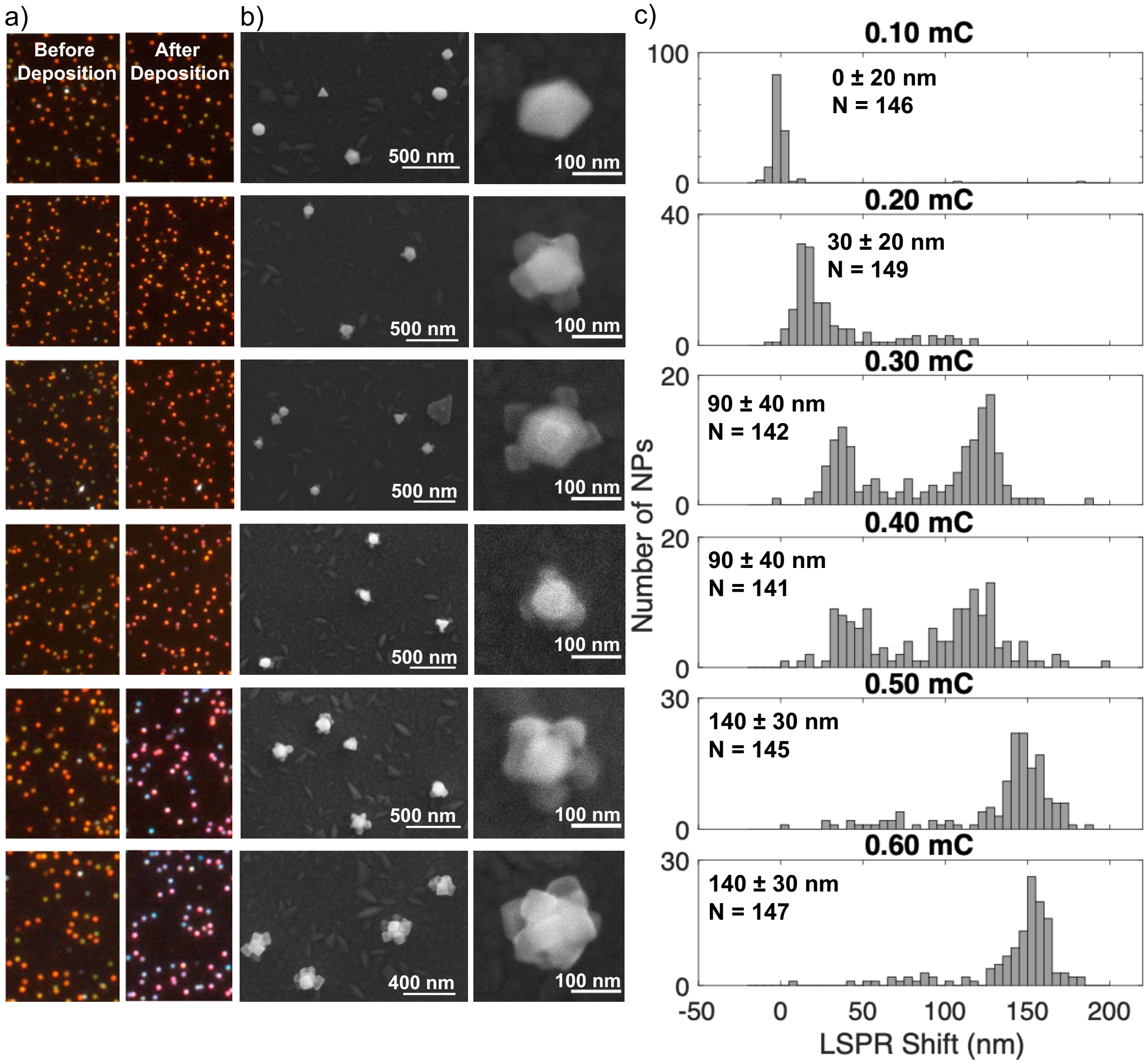

Figure S17. Effect of charge transfer on Cu on Au deposition at a fixed current of 200 μA. a) Dark field optical scattering images from the same region before (left) and after (right) deposition, b) SE SEM images of representative NPs after deposition, and c) LSPR shifts after electrodeposition, with the average, standard deviation and number of NPs (N) reported on each histogram.

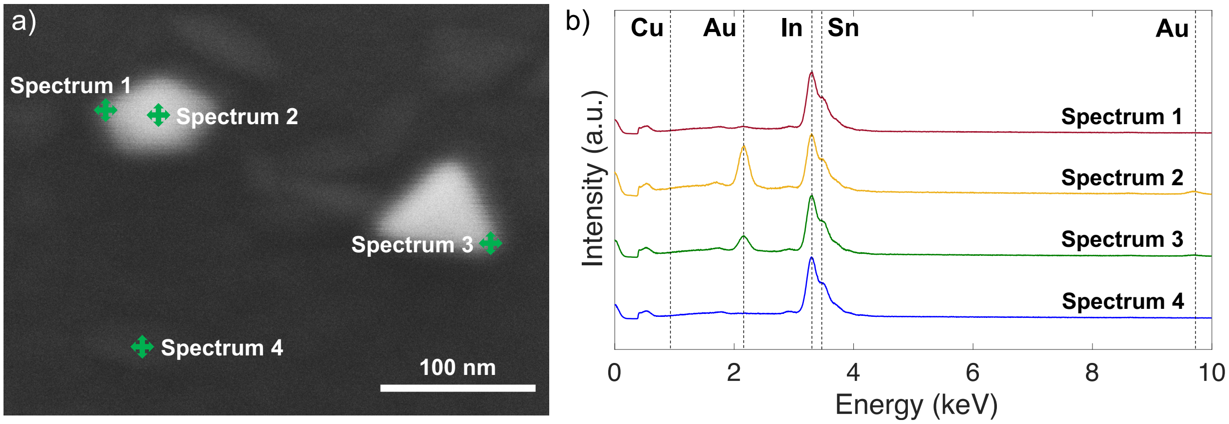

Figure S18. SEM-EDS spectra confirming the absence of Cu. a) SEM image of Au NP after electrodeposition to 0.1 mC of total charge transfer at 100 μA, and b) SEM-EDS spectra of regions selected in a.

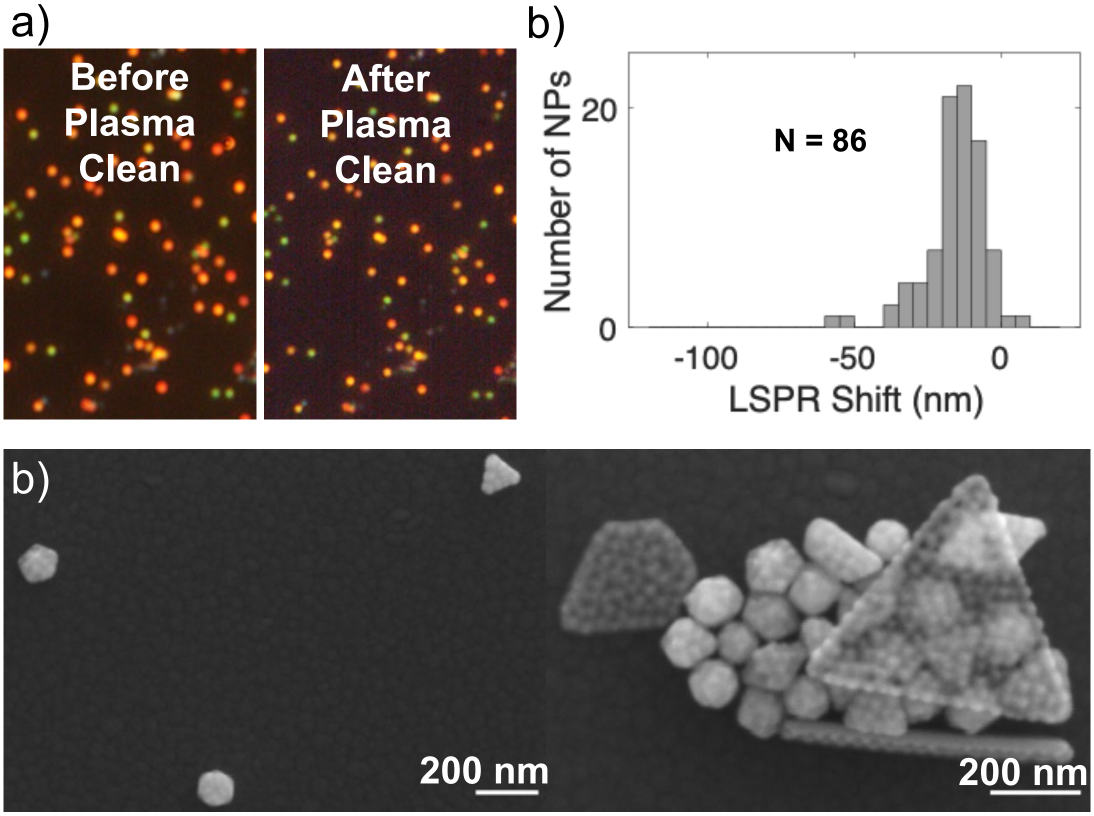

Figure S19. a) Dark field optical scattering images from the same region before (left) and after (right) PVP removal by 5 mins, 20 W Ar/O_2 plasma treatment, b) histogram of LSPR shift, and c) SE SEM images of representative Au NPs after plasma treatment.

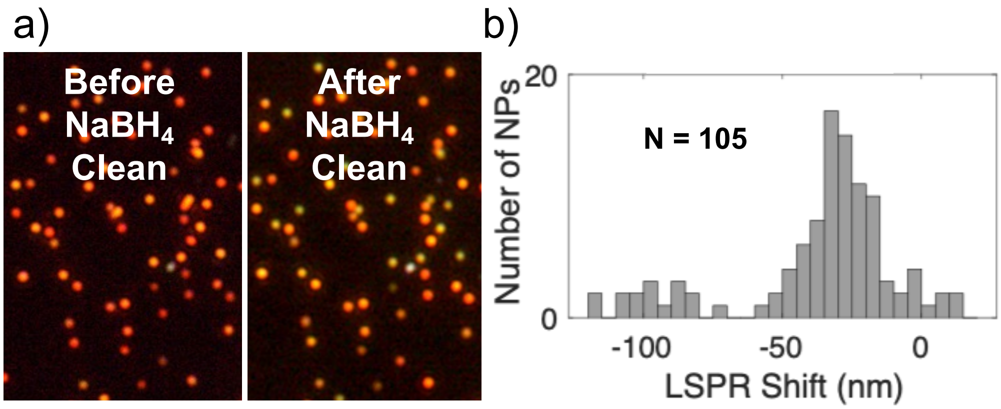

Figure S20. a) Dark field optical scattering images from the same region before (left) and after (right) PVP removal by $NaBH_4$ treatment, and b) histogram of LSPR shift after $NaBH_4$ treatment.

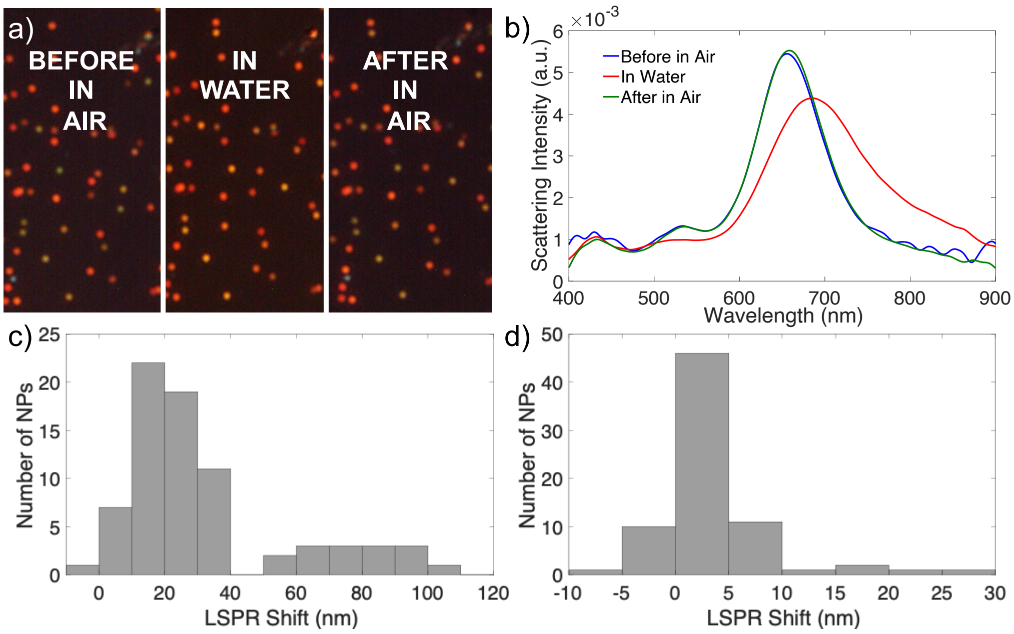

Figure S21. Effect of environment on Au NP LSPR. a) Dark field optical scattering images of Au NPs in air, in aqueous $CuSO_4$ solution and in air again after drying, b) scattering spectra of a single AuNP in air (before/after) and in water, showing a red shift of 29 nm in water, c) histogram of LSPR shift between air and water, and d) histogram of LSPR shift between before and after in air, showing minimal change. N for both histograms is 75.
